# Microbial exposure and host responses to infection reshape the disease trajectory of MASLD

**DOI:** 10.3389/fcimb.2026.1932870

**Published:** 2026-08-20

**Authors:** Yunqi Zhang, Qingqing Xun, Shuyu Liu, Bo Zhuang, Meiyue Zang, Chengwei Tan, Yu Cui, Ruiji Li, Qianqian Zhang

**Affiliations:** 1 School of Clinical Medicine, Jining Medical University, Jining, Shandong, China; 2 School of Pharmacy, Jining Medical University, Jining, Shandong, China

**Keywords:** dysbiosis, gut microbiota, gut–liver axis, host-microbe interactions, immunometabolism, infection, metabolic dysfunction-associated steatotic liver disease, microbial exposure

## Abstract

Metabolic dysfunction-associated steatotic liver disease (MASLD) has become a major driver of the growing global burden of chronic liver disease and has attracted increasing attention because of its progressive hepatic outcomes. Metabolic syndrome-related factors, such as obesity and abnormal glucose metabolism, confer on MASLD both a risk of liver disease progression and systemic metabolic consequences. Evidence from observational studies and experimental models suggests that infection and microbial exposure may be associated with MASLD progression and may modify its clinical course through alterations in the gut–liver axis, enhanced immune activation, and amplification of metabolic inflammation. Meanwhile, the metabolic disturbances associated with MASLD can remodel the intestinal ecological niche and hepatic immune environment, impairing the host capacity to clear and regulate microbial signals and thereby altering infection susceptibility and clinical outcomes. Despite growing interest in infection and microbial dysbiosis in MASLD, most studies remain centered on individual pathogens. A unifying framework linking microbial exposure, host metabolic dysfunction, and inflammatory amplification is still lacking. For most infectious exposures, causal directionality and clinical modifiability remain uncertain. Accordingly, this review organizes the available evidence on the potential bidirectional relationships between infection and MASLD. It examines how gut–liver microbial signals, viral comorbidities, chronic colonization, and acute infection may shape the course of MASLD. It also considers how MASLD-related metabolic dysfunction may, in turn, modify host responses to infection. Overall, incorporating infection and microbial dysbiosis into the conceptual framework of MASLD may help move beyond a metabolism-centered explanatory model and identify priorities for future risk-stratification and intervention studies.

## 1 Introduction

Metabolic dysfunction-associated steatotic liver disease (MASLD) has become one of the most common chronic liver diseases in clinical practice. Over the past three decades, both the prevalence and incidence of MASLD have continued to increase, and its future disease burden may rise further (Kan et al., 2025). The gut–liver axis has become an important direction for exploring therapeutic interventions for MASLD. Recent evidence further indicates that intestinal microbial dysbiosis is not confined to the gut itself. Dysbiosis in extraintestinal niches, such as the oral cavity, may also reshape gut microbial composition and metabolic output, thereby converting local microbial disturbances into liver-sensed inflammatory and lipid-accumulation signals (Chen et al., 2025). Moreover, multi-omics studies have shown that MASLD-related microbial alterations involve not only changes in bacterial communities but also broader transkingdom dysbiosis encompassing fungal and viral components as well as microbial metabolites, suggesting that microbial factors may participate in disease progression through more complex ecological networks (Kim et al., 2025).

As MASLD is increasingly recognized as a complex disease state arising from the interplay among metabolic dysregulation, immune imbalance, and multisystem injury, infection-related factors have emerged as an important lens through which to explain heterogeneity in disease progression and clinical outcomes. Chronic viral infections, long-term pathogen colonization, and responses to acute infection may affect the course of MASLD through persistent hepatic inflammation, low-grade systemic immune activation, and short-term inflammatory stress, respectively, thereby creating a disease context in which infection and metabolic injury overlap. The overlap between MASLD and viral hepatitis is no longer merely a matter of coexisting etiologies, but an important factor that shapes clinical management pathways (Hasanoglu et al., 2025). Studies on acute infection and severe infection outcomes indicate that the presence of MASLD may amplify host clinical responses to infectious stress. Patients with coronavirus disease 2019 (COVID-19) and concomitant MASLD are more likely to develop severe manifestations such as septic shock. Meanwhile, MASLD is associated with an increased risk of severe bacterial infections requiring hospitalization, and this risk further increases with greater fibrosis burden (Sohail et al., 2024; Mantovani et al., 2025). Cohort studies further show that MASLD is associated with an increased risk of incident severe infection, most commonly involving the respiratory and genitourinary tracts. Waist circumference, triglycerides, and hemoglobin A1c (HbA1c) partly mediate this association (Zhao et al., 2025).

The disease trajectory of MASLD is not entirely determined by host metabolic abnormalities, but may be continuously reshaped by the combined effects of intestinal and extraintestinal microbial ecosystems and infection responses. However, current evidence does not yet establish whether these factors act as primary drivers of disease progression or instead modify the clinical course in susceptible individuals. How different microbial exposures converge through the gut–liver axis and immunometabolic networks to influence MASLD progression and infection-related outcomes therefore remains to be clarified. Accordingly, this review examines the interplay among microbial exposure, infection responses, and the host immunometabolic state, while distinguishing clinical associations from experimentally supported mechanisms and hypotheses that require further validation.

## 2 The host defense basis underlying MASLD-associated susceptibility to infection

In the context of chronic inflammation, patients with MASLD undergo systemic remodeling of host defense, which is reflected by increased susceptibility to infection and a higher risk of infection-related adverse outcomes. Studies in MASLD populations have shown that individuals with MASLD are at an increased risk of severe infection, and that this risk rises further with increasing histological severity of liver disease (Ebrahimi et al., 2023; Mantovani et al., 2025).

Under normal physiological conditions, intestinal epithelial tight junctions, the mucus layer, antimicrobial proteins and mucosal immunity together form a multilayered defensive interface that restricts the entry of luminal microorganisms and their metabolic products into the portal venous system (Benedé-Ubieto et al., 2024). In the setting of MASLD, however, increased intestinal barrier permeability, alterations in antimicrobial proteins and endotoxin exposure collectively weaken the capacity to contain luminal microorganisms, thereby allowing pathogen-associated molecular patterns (PAMPs) to more readily enter the portal venous–hepatic circulation (Bergheim and Moreno-Navarrete, 2024). MASLD-associated barrier abnormalities indicate that, even before infection develops, the host capacity to restrict the cross-barrier dissemination of microorganisms and their products has already declined. Specifically, at the level of intestinal mucosal immunity, interleukin-17 (IL-17) derived from small intestinal T helper 17 (Th17) cells helps maintain microbial homeostasis and intestinal barrier integrity. A reduction in small intestinal Th17 cells or impaired IL-17 signaling suppresses the growth of beneficial bacteria and promotes the expansion of pathogenic bacteria, leading to increased endotoxin levels and intestinal mucosal barrier injury. Supplementation with recombinant IL-17, CD4^+^ T cells, or microbiota transplantation from wild-type mice can partially restore barrier integrity (He et al., 2022). Interleukin-22 (IL-22) further links barrier repair to intracellular signaling within intestinal epithelial cells. Obesogenic diets suppress small intestinal IL-22 expression and reduce the activity of signal transducer and activator of transcription 3 (STAT3) in intestinal epithelial cells, resulting in the expansion of absorptive enterocytes and disruption of intestinal homeostasis. Exogenous IL-22 fusion protein acts predominantly through epithelial interleukin-22 receptor subunit alpha 1, activating STAT3 and inhibiting Wnt/β-catenin signaling, thereby reversing both diet-induced abnormal enterocyte expansion and enhanced nutrient absorption (Zhang et al., 2024). At the structural level of the barrier, high-fat diet-induced deglycosylation of intestinal epithelial mucin-1 (MUC1) promotes clathrin-mediated endocytosis and neural precursor cell expressed developmentally down-regulated 4-mediated lysosomal degradation of MUC1, which subsequently induces β-catenin degradation and disrupts the intestinal epithelial barrier (Li et al., 2025). At the level of intestinal epithelial metabolism, loss of intestinal epithelial transmembrane 6 superfamily member 2 (TM6SF2) induces intestinal barrier injury, enrichment of pathogenic bacteria and increased portal venous lysophosphatidic acid by disrupting the interaction between TM6SF2 and fatty acid-binding protein 5. The elevated lysophosphatidic acid then enters the liver through the gut–liver axis, where it promotes lipid deposition and inflammatory responses (Zhang et al., 2025b). The insulin-like growth factor 1 receptor (IGF-1R)/phosphoinositide 3-kinase (PI3K)/protein kinase B (AKT) signaling axis may be involved in intestinal barrier repair in the context of MASLD. In MASLD rats, impaired intestinal barrier function is accompanied by suppression of the growth hormone–IGF-1, as reflected by increased diamine oxidase and D-lactate levels, villous structural damage, and reduced expression of tight junction proteins and mucin-2. In a lipopolysaccharide (LPS)-induced barrier injury model of Caco-2 cells, exogenous IGF-1 increased IGF-1R, PI3K, and AKT expression and promoted epithelial proliferation. It also reduced apoptosis, increased transepithelial electrical resistance, and restored the expression of barrier-related molecules (Zhao et al., 2026). MASLD-associated intestinal barrier injury develops against a background of dysregulated mucosal immune regulation. In this setting, epithelial homeostasis becomes difficult to maintain and is accompanied by structural barrier damage and a reduced capacity for metabolic repair. These changes increase the input of gut-derived pathogen-associated molecules before infection occurs, thereby providing an upstream basis for heightened susceptibility to infection. From this perspective, MASLD-associated intestinal barrier abnormalities represent a state of persistent immune exposure. Chronically elevated input of gut-derived signals places the liver in a pre-activated environment, so that, once infection occurs, the transition from a defensive response to uncontrolled inflammation is more readily triggered.

Kupffer cells are key filtering cells for portal vein-derived microbial signals upon their entry into the liver, and the maintenance of Kupffer cell homeostasis directly determines the efficiency with which the liver captures pathogens within hepatic sinusoids and limits their dissemination. Impaired Kupffer cell positioning can weaken the primary filtration of portal vein-derived pathogens by the liver. Bone morphogenetic protein 9/bone morphogenetic protein 10–activin receptor-like kinase 1 (ALK1)–SMAD family member 4 (SMAD4) signaling maintains Kupffer cell self-renewal, the Clec4F/Tim4 phenotype and V-set and immunoglobulin domain-containing 4 (VSIG4) expression. Loss of ALK1 or SMAD4 leads to a reduction in Kupffer cells and their replacement by monocyte-derived macrophages, accompanied by decreased complement-mediated capture capacity, impaired clearance of blood-borne bacteria and an increased risk of disseminated infection (Zhao et al., 2022). Impaired Kupffer cell homeostasis is also reflected in a decline in protective intercellular communication. Under physiological conditions, Kupffer cell-derived exosomal microRNA-690 (miR-690) suppresses hepatocyte lipogenesis, inflammatory responses in recruited macrophages and fibrogenic activation of hepatic stellate cells (HSCs). During the progression of metabolic dysfunction-associated steatohepatitis (MASH), however, reduced miR-690 expression in Kupffer cells attenuates the repression of nicotinamide adenine dinucleotide kinase (NADK), leading to NADK upregulation and decreased nicotinamide adenine dinucleotide levels, thereby promoting lipid deposition and inflammatory progression (Gao et al., 2022). When gut-derived pathogen-associated and damage-associated molecules continuously enter the liver, their inflammatory consequences also depend on whether negative regulatory control of intrahepatic innate immune signaling remains intact. Once infection-related nucleic acid-sensing pathways are activated, the TANK-binding kinase 1 (TBK1)–interferon regulatory factor 3 (IRF3) axis and its downstream type I interferon response need to be appropriately restrained to prevent anti-infective defense responses from being persistently amplified into chronic inflammation. Hepatocyte nuclear factor 1 alpha (HNF1A) declines during MASLD progression and can inhibit TBK1–IRF3 signaling by promoting the autophagic degradation of TBK1. Loss of HNF1A enhances the TBK1–IRF3 pathway and the type I interferon response, suggesting that insufficient negative regulatory capacity may convert nucleic acid-sensing signals originally involved in anti-infective defense into persistent hepatic inflammation (He et al., 2023). A similar shift in the threshold for nucleic acid sensing also occurs within hepatic macrophages themselves. Activation of the c-Jun N-terminal kinase (JNK)–forkhead box O1 (Foxo1) axis, in cooperation with yes-associated protein (YAP)/Notch1 signaling, upregulates cyclic GMP–AMP synthase (cGAS) expression, thereby enhancing stimulator of interferon genes (STING)–TBK1–nuclear factor κB (NF-κB) signaling and the release of inflammatory mediators. Foxo1 deficiency suppresses this pathway and alleviates inflammation, whereas disruption of YAP or Notch1 reinstates an enhanced STING response (Xu et al., 2024). Together, these findings suggest that, in the setting of MASLD, macrophages exhibit reduced clearance capacity, enhanced inflammatory sensing and insufficient negative regulatory control. As a result, pathogen-associated molecules become more difficult to eliminate but more likely to induce inflammatory amplification, thereby contributing to increased susceptibility to infection.

**Figure 1:**
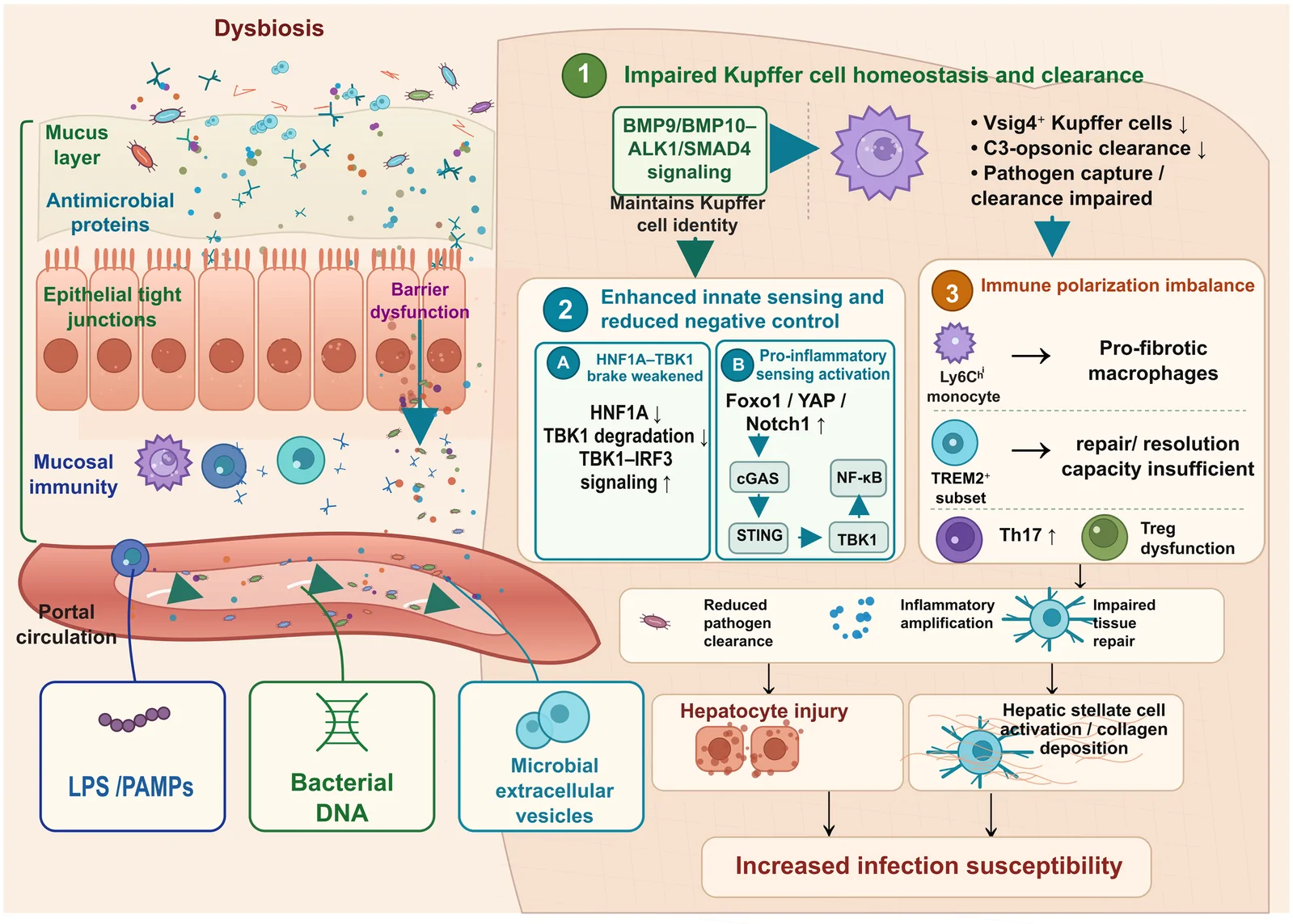
Host defense mismatch underlying MASLD-associated susceptibility to infection. MASLD-related dysbiosis and intestinal barrier dysfunction weaken mucosal defense, allowing gut-derived microbial components, including LPS/PAMPs, bacterial DNA, and EVs, to enter the portal circulation. After reaching the liver, these signals encounter impaired Kupffer cell homeostasis, reduced pathogen clearance, enhanced innate immune sensing, and insufficient negative regulatory control. Meanwhile, polarization shifts in myeloid cells, Th17 cells, and Treg cells further amplify inflammation and limit tissue repair. MASLD, metabolic dysfunction-associated steatotic liver disease; LPS, lipopolysaccharide; PAMPs, pathogen-associated molecular patterns; DNA, deoxyribonucleic acid; EVs, extracellular vesicles; Th17, T helper 17; Treg, regulatory T cell.

An imbalance in immune-cell polarization further explains why patients with MASLD may enter a state characterized by inflammatory amplification but inadequate effective defense after infection. Lipid stress can shape the trajectory of myeloid-cell polarization. Long-chain saturated fatty acids induce the expression of early growth response 2, driving the differentiation of Ly6C^hi monocytes into lipid-associated macrophages with profibrotic features (Iwata et al., 2024). However, the function of lipid-associated macrophages is stage-dependent. During disease regression, the triggering receptor expressed on myeloid cells 2 (TREM2)^+^ subset promotes the resolution of inflammation and fibrosis by enhancing phagocytosis, lipid handling and collagen degradation, whereas insufficient function of this subset limits inflammatory resolution (Ganguly et al., 2024). A central feature of myeloid polarization imbalance in MASLD is the persistent activation of pro-inflammatory and pro-fibrotic programs, together with a relative insufficiency in repair and inflammatory resolution. Skewing of adaptive immunity further amplifies this inflammatory background. Secreted phosphoprotein 1 can activate extracellular signal-regulated kinase signaling by interacting with integrin subunit beta 1 and CD44 on the surface of T cells, thereby promoting Th17 cell differentiation and enhancing IL-17A-related inflammatory responses (Zheng et al., 2024). Meanwhile, regulatory T (Treg) cells may also undergo functional redirection during chronic metabolic injury. Amphiregulin-positive Treg cells are enriched in the MASH liver, and amphiregulin secreted by these cells activates HSCs through epidermal growth factor receptor signaling, thereby promoting fibrosis while aggravating metabolic dysfunction (Savage et al., 2024). These interconnected mechanisms are summarized in **Figure 1**.

MASLD-associated susceptibility to infection may not be adequately explained by either “immunosuppression” or “immune hyperactivation” alone. Instead, current clinical and experimental evidence supports a working model of mismatched host defense. In this model, impaired intestinal barrier containment, altered Kupffer cell and macrophage function, and immune-cell polarization may jointly reduce the efficiency of pathogen clearance while increasing the propensity for inflammatory amplification and tissue injury. However, much of the mechanistic evidence derives from animal or cellular models, and the extent to which these abnormalities directly mediate infection susceptibility in patients with MASLD remains to be established.

## 3 Distinct pathways through which infection and microecological factors influence MASLD progression

Different microecological factors affect MASLD through distinct pathways, and placing them within a single framework may obscure their specific mechanistic features. Therefore, this review classifies these factors into four patterns: human pathogenic viral infection, long-term colonization-related factors, intestinal microecological remodeling, and host responses to acute infection.

### 3.1 Human pathogenic viral infection pattern

When MASLD coexists with viral infection, virus-related effects and host metabolic imbalance interact, causing originally relatively independent pathogenic processes to converge into shared hepatic pathological responses. Virological control does not mean that the risk of liver disease progression has been eliminated, and metabolic abnormalities are not merely a coexisting background (Huang and Liu, 2023; Ejaz et al., 2026). Within this framework, hepatitis B virus (HBV), hepatitis C virus (HCV) and Epstein–Barr virus (EBV) provide different pathological scenarios for understanding how viral infection reshapes the risk structure and hepatic outcomes of MASLD.

#### 3.1.1 HBV–MASLD comorbidity: dissociation between viral phenotype and metabolic risk

Immune-mediated hepatocellular injury and metabolic steatosis may jointly influence hepatic inflammation and fibrosis, but the relationship of steatosis with HBV virological activity and long-term outcomes remains inconsistent. Patients with chronic hepatitis B and concomitant fatty liver disease often have lower hepatitis B e antigen positivity rates and lower viral loads. In some untreated cohorts, this association becomes stronger with increasing hepatic fat accumulation. Steatosis reduces the secretion of hepatitis B surface antigen (HBsAg) and HBV DNA by enhancing endoplasmic reticulum stress, whereas adiponectin, which has anti-steatotic effects, can promote HBV replication. Therefore, with increasing hepatic fat accumulation, adiponectin-related pro-replicative signaling is weakened, which may further explain the inverse relationship between steatosis and HBV activity (Huang and Kao, 2022; Huang and Liu, 2023). In a multicenter retrospective cohort study, following propensity score matching, patients with chronic hepatitis B and concomitant fatty liver disease had a lower 10-year cumulative incidence of cirrhosis than patients without fatty liver disease (10.52% vs. 15.47%), a higher HBsAg clearance rate (16.19% vs. 5.92%), and a lower risk of HCC (Li et al., 2021). These findings suggest that hepatic steatosis may be associated with a less active virological phenotype and, in some cohorts, more favorable liver-related outcomes. However, they do not establish a uniformly protective effect of steatosis across all patients with chronic hepatitis B (Li et al., 2021).

Other studies have reported less favorable long-term outcomes in patients with HBV–MASLD comorbidity. Experimental evidence suggests that hepatic steatosis and metabolic dysfunction may exert partly distinct effects. Steatosis may reduce the secretion of HBsAg and HBV DNA through endoplasmic reticulum stress, lipid-droplet remodeling, and impaired secretory pathways, thereby contributing to a relatively low-replication phenotype. Cardiometabolic abnormalities and lipotoxic metabolites may activate mitochondrial reactive oxygen species (ROS) production, JNK/NF-κB signaling, and the NLR family pyrin domain-containing 3 (NLRP3) inflammasome. In parallel, HBV integration and HBV X protein-related signaling may enhance oxidative stress, DNA damage, and pro-oncogenic transcriptional programs (Zhang et al., 2025a). These mechanisms provide biological plausibility for persistent fibrotic or oncogenic risk despite relatively favorable serum virological markers, but they do not establish that MASLD uniformly worsens long-term outcomes in patients with chronic hepatitis B. Consistent with a potentially adverse effect, one cohort reported a higher incidence of HCC in patients with chronic hepatitis B and concomitant MASLD than in those without MASLD (4.8% vs. 1.2%), and MASLD remained independently associated with HCC after inverse probability weighting (Lin et al., 2024).

The conflicting findings across studies likely reflect heterogeneity in both patient characteristics and study design. Antiviral treatment and the duration of viral suppression may alter the balance between virus-related injury and metabolic stress, while HBV genotype, disease phase, and baseline fibrosis further shape long-term outcomes. The prognostic effect of MASLD may also vary with cardiometabolic burden and the background HCC risk of different populations, which partly accounts for regional differences. Interpretation is further complicated by inconsistent definitions of hepatic steatosis, fatty liver disease and MASLD, as well as variation in the methods used to assess steatosis. Residual confounding remains an additional concern. Importantly, isolated hepatic steatosis may not carry the same prognostic significance as steatosis accompanied by substantial metabolic dysfunction.

Management of HBV–MASLD comorbidity should extend beyond virological control to the continued assessment of residual liver-related risk, as low HBV DNA levels alone may not fully capture the risk of fibrosis progression or HCC development. A dynamic assessment framework integrating HBV replication levels, changes in antigen status, and transaminase indicators remains central to the evaluation of virological activity and treatment response. Non-invasive fibrosis assessments, including liver stiffness and the fibrosis-4 index (FIB-4), can complement these virological measures, together with evaluation of cardiometabolic factors such as diabetes, obesity, and dyslipidemia. In patients with advanced fibrosis, cirrhosis, substantial metabolic abnormalities, or established HCC risk factors, follow-up should emphasize overall liver-related risk and the adequacy of imaging surveillance rather than viral load alone. Obesity and hepatic fat accumulation may impair ultrasound visualization; when image quality is inadequate, cross-sectional imaging may be considered as a complementary surveillance modality. Overall, metabolic abnormalities may represent potentially modifiable contributors to residual fibrosis and HCC risk after virological control and should therefore be considered alongside virological status in risk stratification, fibrosis assessment, and HCC surveillance.

#### 3.1.2 HCV–MASLD comorbidity: residual metabolic risk after viral clearance

HCV and MASLD intersect more directly at the level of lipid metabolism and insulin resistance. HCV replication, assembly and release depend on hepatocyte lipid droplets and the lipoprotein secretory machinery, and the virus itself can alter host lipid handling, thereby inducing hepatic steatosis and insulin resistance (Kralj et al., 2016; Elgretli et al., 2023). In HCV genotype 3 infection, the degree of steatosis often changes with viral load, decreases after viral clearance and reappears with viral relapse, exhibiting virus-driven features that distinguish it from purely metabolic steatosis (Abenavoli et al., 2014). In HCV infection with genotypes other than genotype 3, however, steatosis more often reflects host metabolic dysfunction, such as obesity, insulin resistance and dyslipidemia. Even after HCV clearance, residual or newly emerging metabolic dysfunction may sustain hepatic fat accumulation and contribute to persistent liver-related risk (Kralj et al., 2016).

The use of antiviral therapy has shifted the clinical focus of HCV–MASLD comorbidity. After virological cure, the inflammatory burden driven by HCV replication improves, but the host metabolic state does not necessarily recover in parallel. This is reflected by a shift in the lipid profile from a virus-suppressed state toward the host metabolic baseline, allowing the underlying risk of lipid metabolic abnormalities to re-emerge. After sustained virological response is achieved, total cholesterol and low-density lipoprotein cholesterol (LDL-C) levels may begin to rise at the end of treatment and remain elevated for approximately one year (Mei et al., 2024b). These changes may represent restoration of host lipoprotein metabolism following resolution of HCV-associated hypolipidemia and require interpretation within the broader trajectory of glycemic control, adiposity and hepatic steatosis. A study of patients with chronic hepatitis C who achieved viral eradication showed slight reductions in HbA1c and body mass index (BMI), accompanied by increases in total cholesterol, LDL-C and triglycerides. The prevalence of steatotic liver disease changed from 36.8% to 37.5%, while that of MASLD changed from 34.0% to 34.8%. The stability of these estimates suggests that viral eradication alone has limited impact on the post-cure burden of metabolic steatosis. Higher BMI, LDL-C and HbA1c levels were associated with incident MASLD after viral eradication, suggesting that the post-cure trajectory of hepatic steatosis depends more on host cardiometabolic risk than simply on whether the virus has been cleared (Hui and Mak, 2025). Pangenotypic antiviral studies show a similar dissociation. Among patients who achieved sustained virological response, LDL-C and total cholesterol increased significantly, whereas triglycerides, high-density lipoprotein cholesterol, HbA1c and the homeostasis model assessment of insulin resistance remained unchanged. This divergence separates restoration of circulating lipoprotein levels from recovery of insulin sensitivity (Ko et al., 2025). After virological cure of HCV infection, liver stiffness and FIB-4 often decline during early follow-up. These early reductions partly reflect attenuation of necroinflammation and may overestimate the extent of structural fibrosis regression. Diabetes, obesity and hypercholesterolemia are associated with a lower likelihood of improvement in liver stiffness or FIB-4 (Liu and Chang, 2025), indicating persistent metabolic stress after viral clearance. Post-cure changes in these markers reflect the combined effects of inflammatory resolution, residual fibrosis and ongoing metabolic injury. Residual metabolic risk after virological cure may also affect the occurrence of HCC. Among patients who achieved virological cure and had concomitant MASLD, the 5-year cumulative incidence of *de novo* HCC was significantly higher than that in patients without MASLD (10.4% vs. 3.5%), and MASLD remained associated with an approximately two-fold increased risk of HCC in multivariable analysis (Liu et al., 2025).

Sustained virological response reshapes the relative contributions of viral and metabolic injury to subsequent liver disease progression. Resolution of HCV-driven inflammation and genotype 3-associated steatosis increases the relative contribution of the host cardiometabolic phenotype and pre-existing fibrosis to residual risk. Persistent or newly emerging steatosis after viral clearance reflects heterogeneous processes, including residual metabolic dysfunction, incomplete resolution of hepatic injury, and differences in baseline fibrosis burden. Circulating lipid levels, liver stiffness, and FIB-4 therefore require interpretation within the broader post-cure clinical context. Current evidence supports continued assessment of cardiometabolic and fibrosis-related risk, with HCC surveillance guided by established fibrosis- and cirrhosis-based criteria. The key translational priority is to establish whether modification of metabolic dysfunction after HCV clearance produces durable reductions in fibrosis progression and HCC incidence.

#### 3.1.3 Post-EBV infection state: bile acid perturbation and its association with MASLD risk

Observational studies first linked EBV infection to the risk of MASLD. Patients with infectious mononucleosis had a higher proportion of patients diagnosed with MASLD within 10 years after infection than matched controls (2.64% vs. 1.78%), as well as a higher incidence rate of MASLD (263.9 vs. 164.5 cases per 100,000 person-years). After adjustment for other metabolic factors, infectious mononucleosis remained associated with an increased risk of incident MASLD (adjusted hazard ratio = 1.73) (Loosen et al., 2023). This temporal association remains susceptible to residual metabolic confounding and surveillance bias after infectious mononucleosis. Hepatic involvement during the acute phase of infection provides a more specific pathological entry point for this association, as EBV-related liver injury often occurs in the context of systemic immune activation. Among the children with infectious mononucleosis included in the study, approximately 43.9% had elevated alanine aminotransferase levels, often accompanied by hepatomegaly, splenomegaly, eyelid edema, and increased leukocyte, lymphocyte and atypical lymphocyte counts (Zhang et al., 2021). Among children with concurrent hepatitis and cholestatic liver injury, levels of interleukin-6 (IL-6), tumor necrosis factor-alpha (TNF-α) and soluble intercellular adhesion molecule-1 were higher, and EBV DNA load was also positively correlated with C-reactive protein, TNF-α and counts of multiple immune cell populations. These findings indicate that EBV-related liver injury may be accompanied by concurrent increases in viral burden, inflammatory cytokine release and endothelial adhesion responses (Moppert et al., 2023). Some children with primary EBV hepatitis may present with elevated alkaline phosphatase and γ-glutamyltransferase, suggesting that acute EBV infection can also exhibit a cholestasis-like liver injury phenotype (Yang et al., 2014). Studies of bile acid profiles in infectious mononucleosis provide more direct evidence. Liver injury associated with acute EBV infection is accompanied by remodeling of the bile acid pool composition, showing a characteristic tendency to shift from cholic acid predominance toward enrichment of chenodeoxycholic acid, secondary bile acids and some conjugated bile acids. Glycodeoxycholic acid and glycolithocholic acid showed high predictive value for liver injury in infectious mononucleosis, further suggesting that hydrophobic bile acid subfractions with greater potential hepatotoxicity may serve as more sensitive metabolic signals in EBV infection-related liver injury (Shen et al., 2023). Current evidence defines an acute EBV-associated cholestatic liver phenotype in which immune activation is coupled to remodeling of the bile acid pool. Whether this disturbance persists after recovery or influences subsequent MASLD susceptibility remains unknown.

Remodeling of the bile acid pool after EBV infection and bile acid metabolic reprogramming in MASLD may represent a pathological point of convergence. Bile acid abnormalities are metabolic signals associated with MASH activity and fibrosis risk, and circulating bile acid profiles change with increasing histological severity (Jung et al., 2021). In the context of MASLD, infection-related liver injury and bile acid homeostasis disturbance induced by acute EBV infection may be superimposed on pre-existing abnormalities in bile acid metabolism, further amplifying gut–liver axis imbalance and bile acid metabolic dysregulation. The bile acid axis has become an important therapeutic direction in MASLD. Although there is currently no direct evidence supporting intervention targeting bile acid abnormalities after EBV infection, the bile acid–microbiota axis itself has shown modifiability. In MASLD models, the therapeutic effects of obeticholic acid have been shown to depend on the gut microbiota and to be accompanied by remodeling of the bile acid pool (Liu et al., 2023).

**Figure 2:**
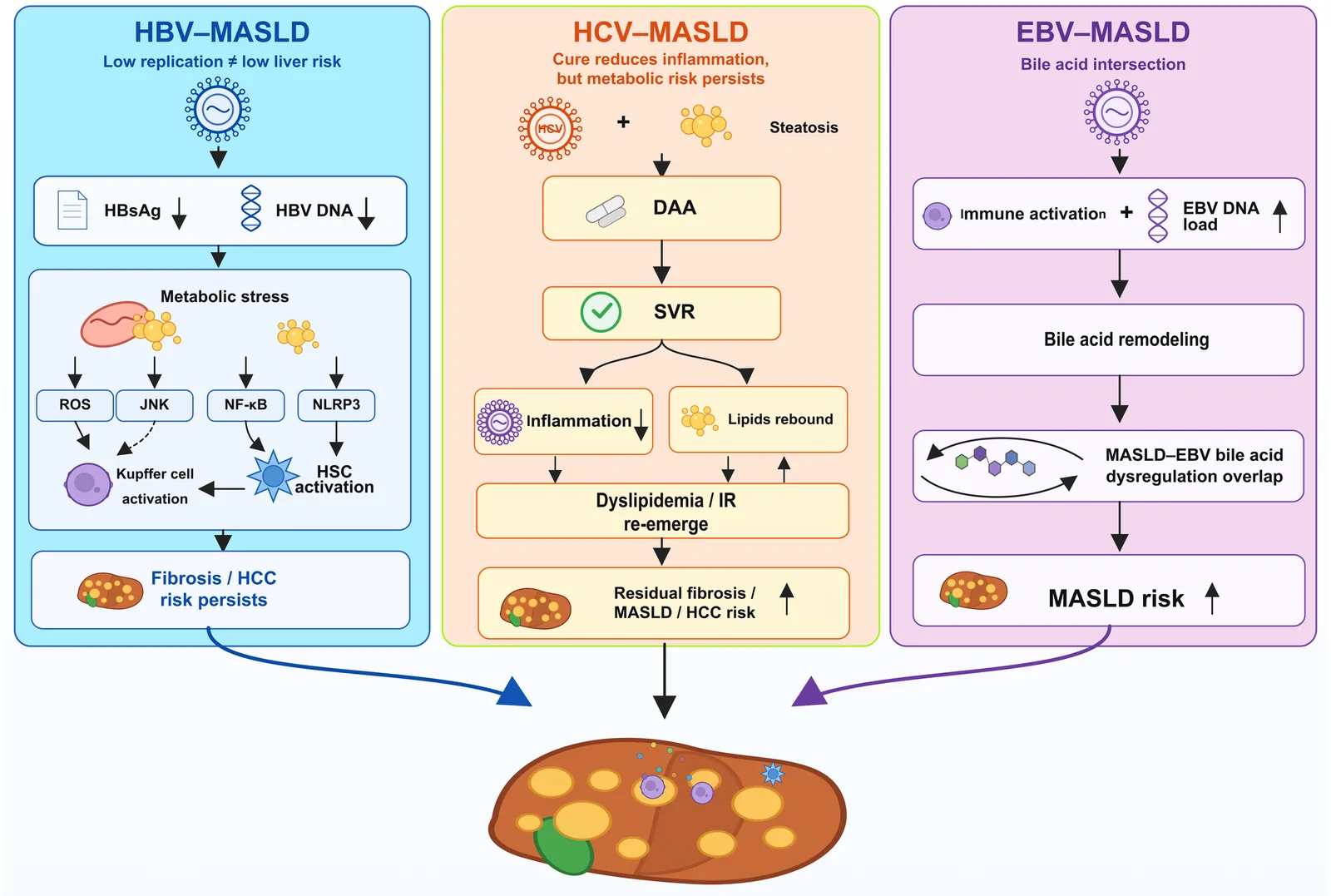
Human pathogenic viral infection patterns shaping metabolic dysfunction-associated steatotic liver disease progression. HBV, HCV, and EBV represent distinct human pathogenic viral infection patterns that may influence MASLD progression through virus-specific but metabolically convergent mechanisms. These include persistent fibrosis and HCC risk despite low HBV replication, residual metabolic risk after HCV clearance, and EBV-related bile acid remodeling. Together, these pathways highlight the convergence of viral infection, metabolic stress, inflammation, and fibrotic progression in MASLD. MASLD, metabolic dysfunction-associated steatotic liver disease; HBV, hepatitis B virus; HCV, hepatitis C virus; EBV, Epstein–Barr virus; HCC, hepatocellular carcinoma; HBsAg, hepatitis B surface antigen; DNA, deoxyribonucleic acid; ROS, reactive oxygen species; JNK, c-Jun N-terminal kinase; NF-κB, nuclear factor kappa B; NLRP3, NLR family pyrin domain containing 3; HSC, hepatic stellate cell; DAA, direct-acting antiviral; SVR, sustained virological response; IR, insulin resistance.

Current evidence suggests a biologically plausible convergence between acute EBV-associated hepatic injury and the bile acid dysregulation characteristic of MASLD. This proposed link is derived from separate epidemiological, acute-phase and experimental datasets, and longitudinal evidence is required to establish whether bile acid remodeling persists after recovery and contributes to subsequent MASLD development or progression. Future studies could establish longitudinal follow-up cohorts of patients with EBV-related liver injury and serially assess bile acid profiles, gut microbiota, hepatic fat content and markers of insulin resistance during the acute infection phase, early recovery phase and within 3–6 months after recovery. This would help determine whether delayed restoration of the bile acid-related microbial and hepatic metabolic axis can predict post-infection MASLD development or progression of pre-existing MASLD. The virus-specific effects of HBV, HCV and EBV on MASLD progression are summarized in **Figure 2**.

### 3.2 Long-term colonization-associated pattern

Among factors related to long-term colonization or persistent infection, the role of *Helicobacter pylori* (*H. pylori*) has long been debated. This controversy is reflected in population-based studies. A prospective cohort study suggested that *H. pylori* infection may be associated with an increased risk of incident MASLD. Among adults without MASLD at baseline, *H. pylori*-positive individuals had a higher risk of developing MASLD during follow-up, and the risk remained increased by approximately 21% after multivariable adjustment (HR 1.21, 95% CI 1.10–1.34) (Kim et al., 2017). Another cross-sectional study showed that *H. pylori*-positive individuals had a higher metabolic burden, including higher BMI, blood pressure, triglycerides and LDL-C, as well as a higher prevalence of MASLD. However, after further adjustment for metabolic factors, the independent association between *H. pylori* and MASLD was no longer observed (OR 0.9, 95% CI 0.9–1.0, P = 0.097) (Fan et al., 2018). Therefore, it remains unclear whether *H. pylori* itself drives MASLD progression or whether it exerts a disease-amplifying effect only in the context of metabolic abnormalities such as obesity and insulin resistance.

Experimental findings tend to support the disease-amplifying effect of *H. pylori*. In mice fed a high-fat diet, *H. pylori* infection accelerated fat accumulation and insulin resistance. This phenotype was accompanied by suppression of insulin receptor substrate 1/AKT signaling, upregulation of the lipogenic factors peroxisome proliferator-activated receptor gamma and CCAAT/enhancer-binding protein beta, and increased expression of TNF-α and interleukin-1 beta (IL-1β). Consistent with these molecular changes, abdominal circumference, visceral fat, fasting insulin levels, and the homeostasis model assessment of insulin resistance were all increased (He et al., 2018). In the context of a high-fat diet, differentially expressed genes in the livers of *H. pylori*-infected mice were mainly enriched in the peroxisome proliferator-activated receptor signaling pathway, fatty acid degradation pathway and retinol metabolism pathway. Increased expression of *sterol regulatory element-binding transcription factor 1*, *fibroblast growth factor 21*, *Il1b* and *Tnf* further suggests the coexistence of lipid metabolic imbalance and inflammatory activation (Chen et al., 2024).

Whether *H. pylori* eradication can translate into sustained clinical benefits in the context of MASLD also remains controversial. A randomized controlled trial showed that, when both groups received lifestyle intervention, patients who underwent *H. pylori* eradication therapy had greater reductions in lipid parameters, insulin resistance and inflammatory markers, but liver function indicators did not show a clear between-group benefit (Yu et al., 2022). A bidirectional Mendelian randomization study provided more cautious evidence from the perspective of genetic causality, finding no clear causal effect of *H. pylori* infection on MASLD in two genome-wide association study datasets for MASLD, and no reverse causal effect of MASLD on *H. pylori* infection (Liu et al., 2022). For patients with MASLD and concomitant *H. pylori* infection, eradication therapy is currently mainly guided by gastrointestinal indications, and *H. pylori* positivity itself is not supported as an independent therapeutic target for MASLD. However, in MASLD patients with concomitant *H. pylori* infection, dynamic follow-up should assess whether the metabolic-inflammatory state, abnormal gut–liver axis inputs and hepatic fat burden improve synchronously after *H. pylori* eradication.

The oral microbiota represents another important direction within the long-term colonization-related pattern. In recent years, research in this area has shifted from describing the comorbidity between periodontitis and MASLD to examining whether oral pathogens can participate in disease progression through the oral–gut–liver axis. In the setting of periodontitis, oral pathogens and their virulence factors, such as LPS, can enter the bloodstream across damaged oral mucosa or reach the intestine through swallowing and participate in gut microbiota remodeling, thereby weakening the intestinal barrier and leading to systemic low-grade inflammation and disordered hepatic lipid metabolism (Mei et al., 2024a). A prospective cohort study suggested that individuals with more severe periodontal attachment loss at baseline were more likely to develop MASLD, and that the risk increased with the extent of periodontal attachment loss. Individuals with attachment loss involving ≥30% of tooth sites had an approximately 60% higher risk of incident MASLD than those without periodontal attachment loss (Akinkugbe et al., 2017). A cross-sectional study suggested that *Porphyromonas gingivalis* (*P. gingivalis*) may represent a key pathogenic signal linking chronic periodontal infection to fibrosis risk in MASLD. Individuals with a higher salivary proportion of *P. gingivalis* had greater liver stiffness measured by magnetic resonance elastography, and a salivary *P. gingivalis* proportion of ≥0.01% was associated with the risk of advanced fibrosis defined by magnetic resonance elastography ≥3.4 kPa (Sato et al., 2022).

Oral pathogens can promote MASLD progression through the oral–gut–liver axis. In a mouse model of oral *P. gingivalis* administration, oral-derived pathogens first entered the intestine through swallowing, causing gut microbiota dysbiosis and inducing Th17/Treg immune imbalance. Subsequently, *P. gingivalis* signals were detectable in the liver, accompanied by a fatty liver-like phenotype, hepatic inflammation and enhanced ferroptosis-related signals (Yao et al., 2023). This axis has been further characterized in a mixed oral microbiota model. Salivary microbiota from patients with periodontitis aggravated intestinal barrier injury in mice fed a high-fat diet, facilitating the transport of LPS to the liver and activation of Toll-like receptor 4 (TLR4) signaling, thereby inducing the release of pro-inflammatory cytokines such as IL-1β. At the same time, the hepatic tryptophan–kynurenine–aryl hydrocarbon receptor axis was upregulated, suggesting that the oral microbiota can convert inflammatory signals originating in the oral cavity into hepatic lipid deposition and disorders of glucose and lipid metabolism (Wang et al., 2023a). Periodontitis-related extracellular vesicles may constitute another distal signaling pathway that regulates hepatocyte lipid metabolism. Exosomes released by macrophages and periodontal ligament fibroblasts activated by *P. gingivalis*-derived LPS can be taken up by hepatocytes, promoting triglyceride synthesis and lipid droplet accumulation by inhibiting AMP-activated protein kinase phosphorylation and upregulating stearoyl-CoA desaturase-1 (Ding et al., 2024). Oral infection induced by *P. gingivalis*, *Treponema denticola*, *Tannerella forsythia* and *Fusobacterium nucleatum* can simultaneously disrupt the oral–gut–liver axis, increasing hepatic bacterial exposure and lipid accumulation, accompanied by changes in genes involved in mitochondrial regulation, oxidative phosphorylation and iron metabolism (Kuraji et al., 2024).

Compared with the gut microbiota, the oral microbiota has more accessible clinical routes for intervention. Current clinical applications mainly involve basic periodontal therapy and plaque control, whose value lies in reducing the pathogenic bacterial burden within periodontal pockets and the local inflammatory burden. Control of oral infectious foci has been associated with improvement in hepatic metabolic phenotypes in some patients, specifically reflected by decreases in endotoxin levels, liver fat content and anti-*P. gingivalis* antibody titers after periodontal treatment (Kamata et al., 2022).

On this basis, oral microbiota intervention should first involve attribution-based stratification before treatment. For patients with MASLD and concomitant periodontitis, salivary or subgingival plaque sequencing can be used to determine whether *P. gingivalis* and red-complex bacteria show dominant colonization. This can be further combined with strain-level source tracking and virulence factor detection to clarify whether oral pathogenic signals have extended to the gut or circulating microecological network. If these oral-derived signals are accompanied by activation of inflammatory pathways along the gut–liver axis and enhanced hepatic metabolic stress, especially manifested as TLR4-mediated inflammatory responses, increased extracellular vesicle-related lipid synthesis or aggravated mitochondrial oxidative stress, these patients may be considered more likely to have a MASLD phenotype with oral microbiota involvement. Such patients may be more suitable candidates for oral microbiota-targeted intervention. Conversely, if there is no evidence that oral-derived signals extend to the gut–liver axis, the goal of periodontal treatment should mainly be limited to local oral benefits and should not be extrapolated as an interventional strategy for concomitant MASLD. Recent studies have shown that nisin can synchronously remodel the oral, gut and hepatic microbiota in a polymicrobial periodontal infection model, reducing hepatic bacterial exposure and lipid deposition. This finding provides clues supporting bacteriocin-based interventions as a potential therapeutic approach targeting oral microbiota-driven MASLD phenotypes (Kuraji et al., 2024).

### 3.3 Gut microbiota remodeling pattern

Gut fungi account for a relatively small proportion of the total gut microbiota, but they can influence the intestinal barrier and hepatic inflammation through fungal-associated molecular patterns, metabolites and induced immune responses (Zeng and Schnabl, 2024). Non-obese patients with MASLD with MASH and F2–F4 fibrosis exhibit distinguishable fecal mycobiome features, characterized mainly by increased ratios of *Mucor* and *Candida albicans* to *Saccharomyces cerevisiae*. Patients with advanced fibrosis show further increases in anti-*Candida albicans* immunoglobulin G levels, suggesting that MASLD progression is accompanied by remodeling of the gut fungal niche and enhanced systemic antifungal immune responses. Animal experiments further showed that antifungal treatment could attenuate the Western diet-induced steatohepatitis phenotype in mice (Demir et al., 2022). Integrated metagenomic and serum metabolomic analyses showed that patients with MASLD exhibited alterations in specific fungal taxa and metabolic networks. Fungi such as *Pseudopithomyces*, *Mucor*, *Aspergillus* and *Ascochyta* were enriched in patients with MASLD. The mycobiome explained 38.2% of the variation in the serum metabolome and was linked to metabolites related to hepatic lipid metabolism, including bile acids, α-linolenic acid and phenylacetic acid. Fungi such as *Alternaria* and *Malassezia* were positioned as central nodes in the altered fungal–bacterial co-abundance network (Zheng et al., 2025). Together, these findings position the gut mycobiome as a potential contributor to MASLD-related metabolic remodeling. Human studies currently delineate disease-associated fungal configurations, while antifungal intervention provides functional support in experimental models. Their temporal stability, causal contribution and reproducibility across MASLD populations remain to be established.

Virome and bacteriophage studies extend MASLD-related microecological imbalance to virus–bacterial host–metabolite ecological remodeling. Virus-like particle sequencing studies have shown that virome features may provide complementary information for MASLD disease stratification. Gut viral diversity and the proportion of bacteriophages decrease with increasing MASLD activity and fibrosis severity, and the performance for identifying F2–F4 fibrosis is further improved when viral diversity is integrated with clinical variables (Lang et al., 2020). These findings suggest that current virome signatures may be more informative as markers of altered microecological stability than as established independent pathogenic factors.

MASLD-related bacteriophage alterations show clear specificity for bacterial hosts and may participate in gut niche remodeling through the selection of bacterial hosts. In the context of hepatic steatosis, *Lactococcus lactis*-related phages were reduced, *Crassvirales* co-occurred with *Prevotella copri*, and *Inoviridae* altered the functional state of host bacteria by affecting processes such as bacterial growth, virulence, biofilm formation and gene transfer (Lang et al., 2020). At the taxonomic level, phages enriched in individuals with MASLD mainly target *Bacteroides*, whereas phages enriched in non-MASLD individuals are more frequently associated with genera related to intestinal metabolic homeostasis, such as *Faecalibacterium*, *Oscillibacter* and *Prevotella*. This suggests that virome alterations may reshape the ecological balance among different bacterial hosts. At the functional level, genes related to recombination and horizontal gene transfer, such as *int* and *recD*, are reduced in the MASLD-associated virome, whereas genes related to host interaction, stress adaptation and viral persistence, such as *xerD*, *dnaK* and *hipB*, are increased (Wang et al., 2025). These observations nominate specific phage–host relationships and functional pathways for mechanistic validation. Direct *in vivo* evidence remains limited, and targeted validation will be essential to determine how phage-mediated ecological control shapes gut-derived metabolic signaling and MASLD heterogeneity.

On this basis, multi-kingdom omics studies link bacteriophage alterations, bacterial host function and hepatic pathological phenotypes in a traceable metabolic chain. In MASLD, the direction of bacteriophage-mediated control is shifted. Reduced lytic pressure on *R. gnavus* amplifies the growth advantage of bile acid-modifying bacteria, whereas reductions in *F. prausnitzii*-associated phages and their auxiliary metabolic genes involved in sulfur amino acid metabolism may weaken the ability of protective commensals to maintain metabolic homeostasis. Ultimately, virus–bacterial interactions are translated into an isodeoxycholic acid signal associated with hepatic fat deposition and hepatocyte ballooning (Zhou et al., 2026). The functional plausibility of phage-targeted modulation is further supported by a separate Klebsiella pneumoniae model. High alcohol-producing *Klebsiella pneumoniae* drives hepatic lipid deposition and inflammatory responses through portal ethanol influx, whereas the lytic phage phiW14 selectively reduces the *Klebsiella pneumoniae* burden, thereby interrupting gut-derived ethanol-induced reprogramming of hepatic glucose and lipid metabolism and reducing the expression of inflammatory and lipogenic genes (Gan et al., 2023). Similar bacterial host selectivity is also observed in individuals with metabolic syndrome, further suggesting that virome remodeling is a common feature of altered gut ecological function under metabolically abnormal conditions. In individuals with metabolic syndrome, phages infecting Streptococcaceae and Bacteroidaceae are enriched, whereas phages infecting Bifidobacteriaceae are reduced, accompanied by changes in functional genes related to temperate phages, including those associated with Candidatus Heliusviridae (de Jonge et al., 2022). These studies delineate a graded evidence landscape for phage involvement in MASLD. Multi-kingdom omics define candidate phage–host–metabolite modules, whereas the phiW14 model provides proof of principle that targeted phage modulation can reshape a defined microbial–metabolic pathway *in vivo*. The translational potential of this framework will depend on direct validation of phage–host specificity, reproducibility across MASLD phenotypes, and demonstration of durable liver-specific benefits in clinically relevant settings.

Current mycobiome and virome studies contribute most directly to disease phenotyping and mechanistic hypothesis generation. Human datasets define cross-kingdom signatures associated with steatohepatitis and fibrosis, whereas experimental studies provide functional support for selected fungal and phage-related pathways. Temporal stability, causal direction and reproducibility across populations remain the principal barriers to translation. Longitudinal sampling, standardized cross-kingdom profiling and direct validation of phage–host–metabolite pathways against liver-specific outcomes will be required to establish their value for risk stratification. By linking a defined microbial driver to selective phage depletion and reversal of hepatic metabolic injury, the high alcohol-producing *Klebsiella pneumoniae*–phiW14 model advances the field from descriptive virome signatures towards a causally anchored intervention framework. From a translational perspective, this model supports a precision-microbiome research paradigm in which a functionally validated pathobiont could define a MASLD endotype, inform patient selection and provide a candidate therapeutic entry point. Prospective human studies will determine whether this mechanistic specificity can be translated into durable liver-related benefit.

**Figure 3:**
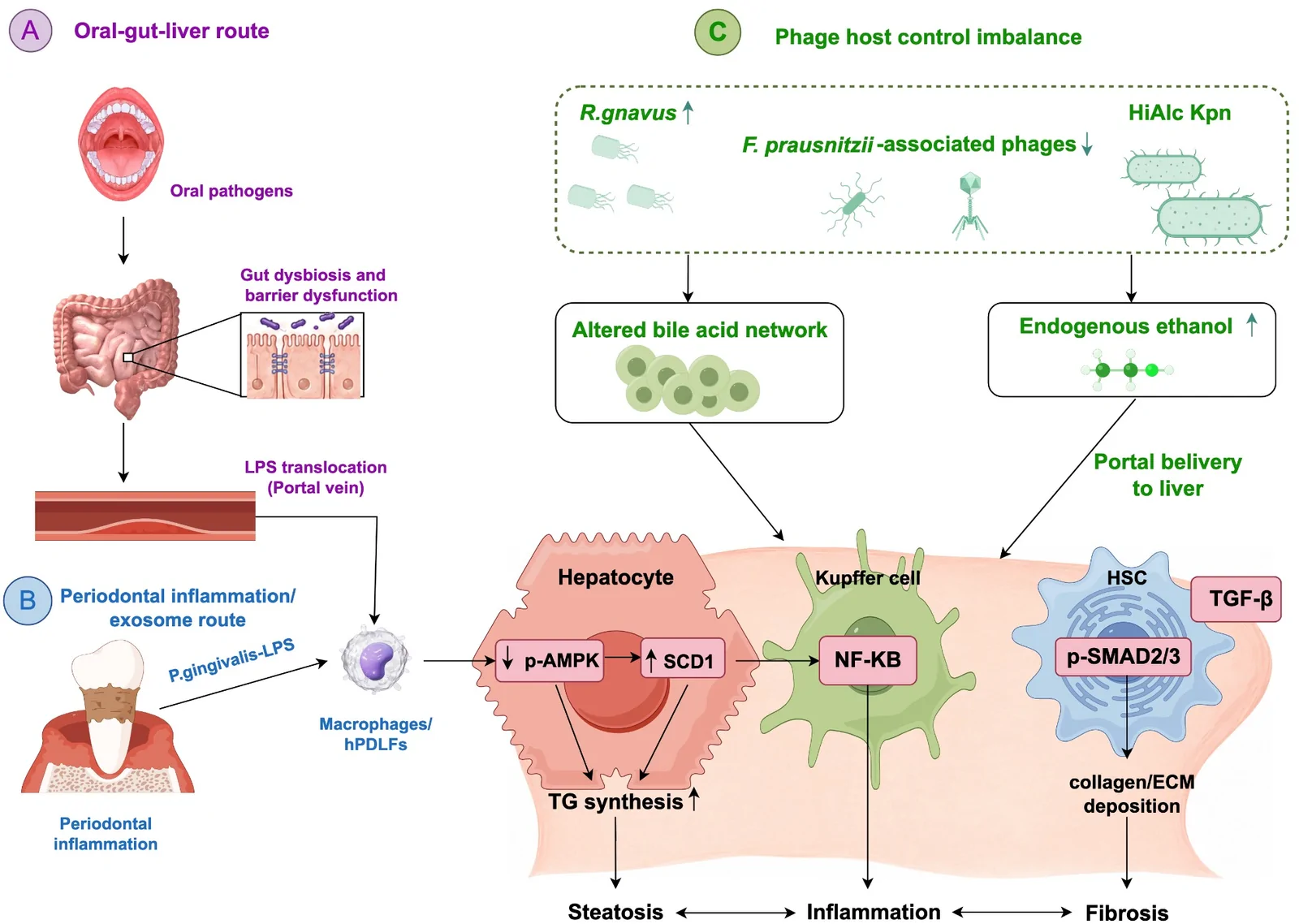
Oral–gut–liver and phage–microbiota pathways linking microbial dysbiosis to MASLD progression. Oral pathogens, periodontitis-derived exosomes, and disrupted phage–host control promote portal microbial signaling, altered bile acid metabolism, and endogenous ethanol production. These pathways converge on hepatocytes, Kupffer cells, and HSCs to drive steatosis, inflammation, and fibrosis. AMPK, AMP-activated protein kinase; ECM, extracellular matrix; HiAlc Kpn, high alcohol-producing Klebsiella pneumoniae; hPDLFs, human periodontal ligament fibroblasts; HSCs, hepatic stellate cells; LPS, lipopolysaccharide; MASLD, metabolic dysfunction-associated steatotic liver disease; NF-κB, nuclear factor kappa B; p-AMPK, phosphorylated AMP-activated protein kinase; p-SMAD2/3, phosphorylated SMAD family member 2/3; SCD1, stearoyl-CoA desaturase 1; TG, triglyceride; TGF-β, transforming growth factor beta.

These mycobiome- and phage-related pathways, together with the oral–gut–liver mechanisms discussed above, are summarized in **Figure 3**.

### 3.4 Acute infection-related host response pattern

Acute infection may serve as a short-term exogenous stressor that amplifies the pre-existing metabolic dysregulation and low-grade inflammatory state in individuals with MASLD. Evidence from studies of COVID-19 suggests that this amplification may reveal an independent immunometabolic risk in younger individuals. A meta-analysis based on multivariable-adjusted effect estimates showed that MASLD was independently associated with an increased risk of severe COVID-19, and that this association was mainly observed in patients younger than 60 years (Wang et al., 2023b). The excess severe-disease risk was mainly reflected by greater disease complexity and a higher burden of respiratory support, rather than by a simple increase in mortality. An analysis of hospitalized patients with COVID-19 showed that, after matching and weighted adjustment for age, sex, race, and other metabolic comorbidities, patients with MASLD still had higher FIB-4 scores and longer hospital stays, whereas in-hospital mortality was not significantly increased (Brozat et al., 2024). These observations may be explained by the presence of MASLD, which skews the inflammatory response during COVID-19 toward an imbalanced state characterized by enhanced pro-inflammatory chemokine/chemotactic signaling and insufficient antiviral immune signaling. Among patients with severe COVID-19 and comparable baseline severity, those with concomitant MASLD exhibited a stronger acute-phase response, greater hepatocellular injury, and an inflammatory profile characterized by increased IL-6, interleukin-8, and C-X-C motif chemokine ligand 10 levels, together with reduced interferon-γ levels. These findings suggest that the acute infectious response in the context of MASLD may be more prone to inflammatory amplification, whereas effective antiviral immune signaling is relatively insufficient (Papic et al., 2022).

COVID-19 data position MASLD as a context-dependent modifier of disease complexity rather than a uniform determinant of mortality. The stronger risk signal observed in younger cohorts suggests that the relative contribution of MASLD may be more discernible when competing age-related determinants of severe disease are less dominant. Greater requirements for respiratory support and prolonged hospitalization, despite inconsistent mortality signals, further indicate that MASLD may shape specific phases and phenotypes of the host response. Cytokine alterations and acute elevations in FIB-4 provide biologically informative signals, although their mechanistic interpretation remains constrained by acute-illness effects and residual cardiometabolic confounding. This heterogeneity provides a clinically relevant framework for combining baseline metabolic and fibrosis phenotyping with longitudinal immune profiling to identify MASLD endotypes that are particularly susceptible to infection-triggered inflammatory amplification and to determine whether these endotypes predict treatment response or post-infectious liver injury.

The acute infectious response pattern may also extend to other serious bacterial infections. MASLD-related metabolic dysregulation may reduce the host capacity to buffer infection-associated inflammation and may thereby contribute to susceptibility to serious infections and adverse outcomes (Targher et al., 2025). Experimental studies suggest that, before an additional acute infectious stimulus, Kupffer cells and macrophages in MASLD models may already exhibit a pro-inflammatory state that is susceptible to further amplification by PAMPs or damage-associated molecular patterns. Saturated fatty acids promote lipid uptake and the formation of foam-like macrophages through macrophage scavenger receptor 1 (MSR1), and induce the release of inflammatory mediators via JNK signaling. Blockade of MSR1 attenuates lipid-induced inflammatory responses in macrophages (Govaere et al., 2022). Against this background, acute stimulation can drive this low-grade inflammation toward a myeloid-cell amplification response. A MASH-inducing diet combined with acute injury can activate the NLRP3 inflammasome and induce the release of IL-1β. In turn, IL-1β acts on hepatocytes to promote the production of C-X-C motif chemokine ligand 1 (CXCL1) and lipocalin-2 (LCN2), thereby driving neutrophil recruitment and the formation of neutrophil extracellular traps. These findings suggest that the NLRP3–IL-1β–CXCL1/LCN2 axis represents an important pathway for acute inflammatory amplification in the context of metabolic inflammation (Babuta et al., 2024). Across experimental systems, metabolically primed macrophages and the NLRP3–IL-1β–CXCL1/LCN2 cascade support a threshold-lowering model in which MASLD amplifies, rather than initiates, the myeloid response to acute infection. These findings do not establish that this pathway is infection specific or uniformly pathogenic, because inflammasome activation and neutrophil recruitment may also contribute to antimicrobial defense, with their net effects likely determined by pathogen type, timing and baseline liver disease severity. The translational question is therefore not whether this axis should be broadly suppressed, but which MASLD endotypes and stages of infection exhibit maladaptive activation that can be safely targeted without compromising pathogen clearance. Because current evidence derives predominantly from diet-induced MASH and acute-injury models, this framework requires validation in pathogen-resolved human cohorts.

**Figure 4:**
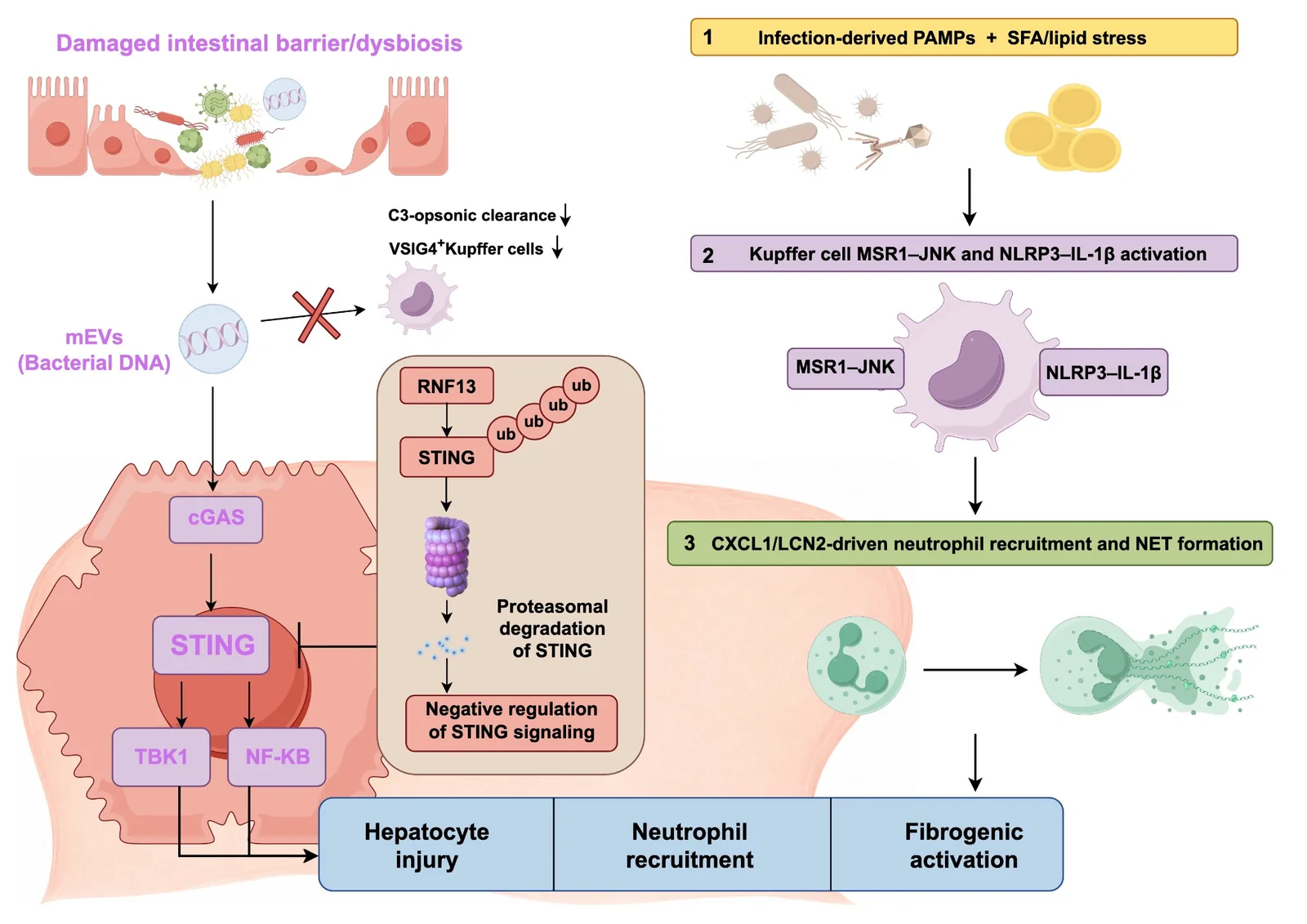
Acute infection-related host responses amplify inflammatory and fibrogenic injury in MASLD. Infection-derived PAMPs and SFA-induced lipid stress activate Kupffer-cell MSR1–JNK and NLRP3–IL-1β signaling, driving CXCL1/LCN2-mediated neutrophil recruitment and NET formation. In parallel, impaired VSIG4^+^ Kupffer-cell clearance promotes bacterial DNA–cGAS–STING signaling, whereas RNF13-dependent ubiquitination restrains STING activation. C3, complement component 3; cGAS, cyclic GMP–AMP synthase; CXCL1, C-X-C motif chemokine ligand 1; IL-1β, interleukin-1 beta; JNK, c-Jun N-terminal kinase; LCN2, lipocalin 2; MASLD, metabolic dysfunction-associated steatotic liver disease; mEVs, microbial extracellular vesicles; MSR1, macrophage scavenger receptor 1; NETs, neutrophil extracellular traps; NF-κB, nuclear factor kappa B; NLRP3, NLR family pyrin domain containing 3; PAMPs, pathogen-associated molecular patterns; RNF13, ring finger protein 13; SFA, saturated fatty acids; STING, stimulator of interferon genes; TBK1, TANK-binding kinase 1; Ub, ubiquitin; VSIG4+, V-set and immunoglobulin domain-containing 4.

In addition to inflammatory pre-activation, MASLD may also impair the hepatic capacity to clear infection-associated molecules and negatively regulate responses to these molecules. During MASH, VSIG4^+^ hepatic macrophages are reduced, and complement component 3 (C3)-mediated opsonic clearance is impaired. As a result, gut-derived extracellular vesicles carrying microbial DNA are more likely to enter hepatocytes and HSCs, thereby activating hepatocellular inflammation and HSC fibrogenic programs through the cGAS–STING pathway (Luo et al., 2022). Thus, STING has dual significance in MASLD. Although it serves as an important pathway for host sensing of pathogen-derived nucleic acids, in the context of MASLD it becomes a key node through which infection-associated molecules amplify hepatic inflammation and fibrosis. In hepatocytes, ring finger protein 13 (RNF13) promotes proteasomal degradation of STING through K48-linked ubiquitination, thereby limiting STING-mediated TBK1/NF-κB inflammatory signaling. When RNF13 is deficient, diet-induced insulin resistance, steatosis, inflammation, cellular injury, and fibrosis are all aggravated, whereas RNF13 overexpression exerts protective effects (Lin et al., 2023). These preclinical studies support a two-step amplification model of MASLD-related liver injury. Loss of VSIG4^+^ macrophage-mediated clearance increases hepatic exposure to microbial nucleic acids. Reduced RNF13-dependent restraint then sustains STING–TBK1/NF-κB signaling in hepatocytes and HSCs. Excessive signal input combined with inadequate intracellular control may convert transient infection-associated cues into persistent hepatic inflammation and fibrogenic activation. The therapeutic implication is selective correction rather than broad STING inhibition. STING remains essential for antimicrobial nucleic-acid sensing, and indiscriminate blockade during active infection may weaken pathogen control. Future studies should identify the MASLD stages and infection contexts in which STING signaling becomes maladaptive and determine whether microbial-product clearance or endogenous pathway restraint can be restored without compromising host defense. Validation across pathogen types and human cohorts remains essential. These acute infection-related host-response pathways are summarized in **Figure 4**.

Current evidence supports acute infection as a plausible amplifier of pre-existing immunometabolic vulnerability in MASLD. The strongest human evidence is derived from COVID-19 cohorts, whereas most pathway-level evidence remains preclinical. Direct evidence across other pathogens and evidence that these pathways are clinically targetable remain limited. Early, guideline-directed treatment of the underlying infection remains the established clinical approach. In a small single-center prospective cohort of patients with MASLD and COVID-19, nirmatrelvir–ritonavir use was associated with a shorter hospital stay and lower fibrinogen levels at discharge (Buchynskyi et al., 2024). These findings support a potential clinical benefit of antiviral treatment in eligible patients, but do not establish a MASLD-specific treatment effect or demonstrate that the observed benefit is mediated by attenuation of infection-related inflammatory amplification. Prospective studies are needed to determine whether MASLD modifies treatment response and whether infection-triggered inflammatory pathways provide actionable targets after pathogen control.

## 4 Discussion

Infection and microbial exposure provide an important entry point for explaining disease heterogeneity and differences in infection-related outcomes in MASLD. Incorporating these factors into MASLD risk stratification and intervention strategies may complement current management models centered on metabolism and fibrosis. Gut-derived microbial products, viral comorbidities, long-term pathogen colonization, and acute infectious stress may influence MASLD progression through distinct pathways. However, current evidence remains insufficient to translate these factors into robust clinical stratification markers or actionable targets. A major obstacle is that infection-related factors are intertwined with the pre-existing immunometabolic imbalance in MASLD, making it difficult to directly infer their true pathological roles from associations with a single pathogen. Accordingly, most infection-related exposures are currently better regarded as candidate, context-dependent disease modifiers rather than established upstream drivers of MASLD. Causal roles for specific pathogens or microbial signals remain plausible but require longitudinal and interventional validation. Existing studies have mostly focused on associations between individual microbial factors and MASLD phenotypes, which makes it difficult to determine whether these changes represent upstream drivers of disease progression, ecological consequences of metabolic dysregulation, or intermediate links in inflammatory amplification at specific disease stages. Microbiome research has expanded beyond the bacterial microbiome to include the mycobiome, virome, and microbial metabolites, but these omics layers are often analyzed separately, and consistent relationships between cross-kingdom ecological networks and hepatic pathological endpoints remain lacking. In infection-related studies, chronic viral comorbidities, long-term pathogen colonization, and acute infectious stress are often grouped within the same infection framework; however, these three types of exposure differ fundamentally in their time scales and host responses, making it difficult to define their specific contributions to MASLD progression. **Table 1** provides an evidence-calibrated synthesis of the NHMRC evidence ranges, principal mechanistic pathways, clinical relevance, and key limitations across the major pathogen- and exposure-specific domains considered in this review.

**Table 1:** Pathogen- or exposure-associated mechanisms, clinical relevance and key limitations in MASLD.

Pathogen or exposure	NHMRC evidence range	Principal mechanistic pathways	Clinical relevance	Key limitations
Hepatitis B virus	Level II–IV	1. Steatosis-induced endoplasmic reticulum stress, lipid-droplet remodeling and impaired secretory pathways may reduce HBsAg and HBV DNA release. 2. Lipotoxic metabolites activate the mitochondrial ROS–JNK/NF-κB–NLRP3 axis. 3. HBV integration and HBV X protein-related signaling promote DNA damage and pro-oncogenic programs.	1. Integrated virological, fibrosis and metabolic risk assessment. 2. HCC surveillance according to established criteria.	1. Conflicting cohort findings. 2. Heterogeneity in antiviral treatment, viral phase and metabolic burden.
Hepatitis C virus	Level II–IV	1. HCV dependence on hepatocyte lipid droplets and lipoprotein secretion promotes steatosis and insulin resistance. 2. After viral clearance, resolution of virus-driven injury increases the relative contribution of residual adiposity, insulin resistance, dyslipidemia and pre-existing fibrosis.	1. Post-SVR metabolic and fibrosis reassessment. 2. HCC surveillance guided by fibrosis stage.	1. Biomarker changes confounded by inflammatory resolution. 2. Baseline fibrosis and metabolic heterogeneity.
Epstein–Barr virus	Level III-2–IV	1. Acute EBV-associated immune activation is coupled to cholestatic liver injury and remodeling of the bile acid pool. 2. If sustained after recovery, bile acid disturbance may converge with pre-existing bile acid dysregulation in MASLD.	1. No established EBV-specific intervention. 2. Longitudinal bile acid profiling remains investigational.	1. Indirect evidence. 2. Residual confounding. 3. Absence of post-recovery longitudinal data.
*Helicobacter pylori*	Aetiology: Level II–IV Intervention: Level II–IV	1. *H. pylori* may suppress insulin receptor substrate 1/AKT signaling and activate PPARγ–CCAAT/enhancer-binding protein β and SREBP1-dependent lipogenic programs. 2. These metabolic changes are accompanied by IL-1β- and TNF-α-mediated inflammation.	1. Eradication according to standard gastrointestinal indications, not MASLD status alone.	1. Discordant epidemiological and genetic evidence. 2. Uncertain liver-specific benefit.
Periodontitis and oral pathogens	Aetiology: Level II–IV Intervention: Level III-2–IV	1. Oral pathogen translocation promotes gut dysbiosis, barrier disruption, Th17/Treg imbalance and LPS–TLR4–IL-1β signaling. 2. Periodontal extracellular vesicles and metabolic reprogramming further engage the AMPK–stearoyl-CoA desaturase-1 and tryptophan–kynurenine–aryl hydrocarbon receptor axes, promoting hepatic lipid accumulation and injury.	1. Standard periodontal care. 2. MASLD-directed microbial stratification remains investigational.	1. Small intervention studies. 2. Limited strain-resolved tracking across the oral–gut–liver axis.
Gut mycobiome	Level IV	1. Fungal-associated molecular patterns and antifungal immune responses may impair intestinal barrier homeostasis and promote hepatic inflammation. 2. Fungal–bacterial ecological networks are linked to remodeling of bile acids and other lipid-related metabolites.	1. Candidate phenotyping tool and mechanistic target. 2. No current basis for antifungal therapy.	1. Predominantly cross-sectional evidence. 2. Uncertain causality, stability and reproducibility.
Gut virome and bacteriophages	Level IV	1. Altered phage host selection and lytic pressure reshape bacterial niches and microbial metabolic output, including bile acid-related pathways. 2. In the high alcohol-producing *Klebsiella pneumoniae* model, portal ethanol drives hepatic metabolic reprogramming, whereas phiW14 interrupts this gut microbial–ethanol pathway.	1. Potential disease stratification and precision-phage framework. 2. Not clinically established.	1. Predicted host assignments. 2. Limited *in vivo* validation. 3. Absence of human efficacy data.
Acute infection	Level III-2–IV	1. MASLD-related lipid stress primes macrophages through MSR1–JNK signaling. 2. The NLRP3–IL-1β–CXCL1/LCN2 axis promotes neutrophil recruitment and inflammatory amplification. 3. Reduced VSIG4^+^/C3-mediated clearance and insufficient RNF13-dependent restraint enhance microbial DNA–cGAS–STING–TBK1/NF-κB signaling in hepatocytes and hepatic stellate cells.	1. Guideline-directed anti-infective care. 2. Immune-pathway targeting remains experimental.	1. COVID-19-dominant human evidence. 2. Pathogen heterogeneity. 3. Predominantly preclinical mechanisms.

AKT, protein kinase B; AMPK, AMP-activated protein kinase; C3, complement component 3; cGAS, cyclic GMP–AMP synthase; CXCL1, C-X-C motif chemokine ligand 1; EBV, Epstein–Barr virus; HBsAg, hepatitis B surface antigen; HBV, hepatitis B virus; HCC, hepatocellular carcinoma; HCV, hepatitis C virus; IL-1β, interleukin-1β; JNK, c-Jun N-terminal kinase; LCN2, lipocalin 2; LPS, lipopolysaccharide; MASLD, metabolic dysfunction-associated steatotic liver disease; MSR1, macrophage scavenger receptor 1; NF-κB, nuclear factor-κB; NLRP3, NOD-, LRR- and pyrin domain-containing protein 3; PPARγ, peroxisome proliferator-activated receptor γ; RNF13, ring finger protein 13; ROS, reactive oxygen species; SREBP1, sterol regulatory element-binding protein 1; STING, stimulator of interferon genes; SVR, sustained virological response; TBK1, TANK-binding kinase 1; Th17, T helper 17; TLR4, Toll-like receptor 4; TNF-α, tumor necrosis factor-α; Treg, regulatory T cell; VSIG4^+^, V-set and immunoglobulin domain-containing 4^+^.

In recent years, research on infection and microecological factors in MASLD has gradually moved toward functional interpretation. Microbiome studies have expanded from a focus on dysbiosis within the gut microbiota to the connections between extraintestinal ecological niches, such as the oral cavity, and the gut microbiome (Sato et al., 2025). Methodologically, multi-omics strategies, including metagenomics, metatranscriptomics, and metabolomics, are driving the field beyond comparisons of microbial abundance toward integrated analyses of microbial composition, functional activity, and metabolic outputs (Kim et al., 2025). Combining source tracking, cross-kingdom interaction analysis, and multi-omics integration models may better enable the identification of key microecological pathways with pathological relevance. Future longitudinal cohorts should combine repeated within-patient sampling with integrated assessment of microbial sources and functional outputs, host inflammatory responses, and non-invasive hepatic phenotypes, while tracking hepatic fat burden, fibrosis progression, and infection-related outcomes. This design would help establish when microecological alterations emerge during MASLD progression and whether they act as upstream drivers, intermediate amplifiers, or downstream consequences of disease. At the level of infectious exposure, the field should move beyond a general discussion of infection-related factors toward distinguishing different patterns of infectious exposure and their corresponding host-response pathways. Refining infection classification along the axes of exposure duration and host-response characteristics would allow different infectious exposures to enter appropriate mechanistic validation frameworks. Chronic viral comorbidities should be evaluated in relation to residual metabolic and liver-related risk after virological control. Long-term colonization studies should examine niche dynamics and gut–liver signaling before and after intervention. Acute infection studies should define how the pre-infection host state shapes post-infection inflammatory amplification. At present, infection and microbial exposure are best viewed as candidate disease modifiers whose mechanistic directionality and interventional relevance require validation in longitudinal and appropriately controlled studies.

## References

[B1] Abenavoli L. Masarone M. Peta V. Milic N. Kobyliak N. Rouabhia S. . (2014). Insulin resistance and liver steatosis in chronic hepatitis C infection genotype 3. World J. Gastroenterol. 20, 15233–15240. doi: 10.3748/wjg.v20.i41.15233 25386071 PMC4223256

[B2] Akinkugbe A. A. Slade G. D. Barritt A. S. Cole S. R. Offenbacher S. Petersmann A. . (2017). Periodontitis and non-alcoholic fatty liver disease, a population-based cohort investigation in the study of health in Pomerania. J. Clin. Periodontol. 44, 1077–1087. doi: 10.1111/jcpe.12800 28804947 PMC5650532

[B3] Babuta M. Nagesh P. T. Datta A. A. Remotti V. Zhuang Y. Mehta J. . (2024). Combined insults of a MASH diet and alcohol binges activate intercellular communication and neutrophil recruitment via the NLRP3-IL-1β axis in the liver. Cells 13, 960. doi: 10.3390/cells13110960 38891092 PMC11171595

[B4] Benedé-Ubieto R. Cubero F. J. Nevzorova Y. A. (2024). Breaking the barriers: the role of gut homeostasis in metabolic-associated steatotic liver disease (MASLD). Gut Microbes 16, 2331460. doi: 10.1080/19490976.2024.2331460 38512763 PMC10962615

[B5] Bergheim I. Moreno-Navarrete J. M. (2024). The relevance of intestinal barrier dysfunction, antimicrobial proteins and bacterial endotoxin in metabolic dysfunction-associated steatotic liver disease. Eur. J. Clin. Invest. 54, e14224. doi: 10.1111/eci.14224 38634717

[B6] Brozat J. F. Ntanios F. Malhotra D. Dagenais S. Katchiuri N. Emir B. . (2024). NAFLD and NASH are obesity-independent risk factors in COVID-19: Matched real-world results from the large PINC AI™ Healthcare Database. Liver Int. 44, 715–722. doi: 10.1111/liv.15815 38110709

[B7] Buchynskyi M. Oksenych V. Kamyshna I. Kamyshnyi O. (2024). Exploring Paxlovid efficacy in COVID-19 patients with MAFLD: Insights from a single-center prospective cohort study. Viruses 16, 112. doi: 10.3390/v16010112 38257811 PMC10819977

[B8] Chen R. Qian J. Wang Q. Li Y. Xu Z. Zhang M. . (2025). Periodontitis exacerbates metabolic dysfunction-associated steatotic liver disease via the gut microbiota-derived tryptophan metabolism-AHR axis in obesity. EBioMedicine 122, 106037. doi: 10.1016/j.ebiom.2025.106037 41274019 PMC12681522

[B9] Chen X. Peng R. Peng D. Liu D. Li R. (2024). Helicobacter pylori infection exacerbates metabolic dysfunction-associated steatotic liver disease through lipid metabolic pathways: a transcriptomic study. J. Transl. Med. 22, 701. doi: 10.1186/s12967-024-05506-y 39075482 PMC11288106

[B10] de Jonge P. A. Wortelboer K. Scheithauer T. P. M. van den Born B. H. Zwinderman A. H. Nobrega F. L. . (2022). Gut virome profiling identifies a widespread bacteriophage family associated with metabolic syndrome. Nat. Commun. 13, 3594. doi: 10.1038/s41467-022-31390-5 35739117 PMC9226167

[B11] Demir M. Lang S. Hartmann P. Duan Y. Martin A. Miyamoto Y. . (2022). The fecal mycobiome in non-alcoholic fatty liver disease. J. Hepatol. 76, 788–799. doi: 10.1016/j.jhep.2021.11.029 34896404 PMC8981795

[B12] Ding C. Shen Z. Xu R. Liu Y. Xu M. Fan C. . (2024). Exosomes derived from periodontitis induce hepatic steatosis through the SCD-1/AMPK signaling pathway. Biochim. Biophys. Acta Mol. Basis Dis. 1870, 167343. doi: 10.1016/j.bbadis.2024.167343 38986822

[B13] Ebrahimi F. Simon T. G. Hagström H. Söderling J. Wester A. Roelstraete B. . (2023). Risk of severe infection in patients with biopsy-proven nonalcoholic fatty liver disease - a population-based cohort study. Clin. Gastroenterol. Hepatol. 21, 3346–3355.e3319. doi: 10.1016/j.cgh.2023.05.013 37245712

[B14] Ejaz M. Kumar K. Sohail D. Sarfaraz I. Azam H. Yaseen F. (2026). MASLD and viral hepatitis overlap: An emerging dual burden in chronic liver disease. Hepatol. Forum 7, 69–76. doi: 10.14744/hf.2025.88386 41589204 PMC12831982

[B15] Elgretli W. Chen T. Kronfli N. Sebastiani G. (2023). Hepatitis C virus-lipid interplay: Pathogenesis and clinical impact. Biomedicines 11, 271. doi: 10.3390/biomedicines11020271 36830808 PMC9953247

[B16] Fan N. Peng L. Xia Z. Zhang L. Wang Y. Peng Y. (2018). Helicobacter pylori infection is not associated with non-alcoholic fatty liver disease: A cross-sectional study in China. Front. Microbiol. 9, 73. doi: 10.3389/fmicb.2018.00073 29445363 PMC5797778

[B17] Gan L. Feng Y. Du B. Fu H. Tian Z. Xue G. . (2023). Bacteriophage targeting microbiota alleviates non-alcoholic fatty liver disease induced by high alcohol-producing Klebsiella pneumoniae. Nat. Commun. 14, 3215. doi: 10.1038/s41467-023-39028-w 37270557 PMC10239455

[B18] Ganguly S. Rosenthal S. B. Ishizuka K. Troutman T. D. Rohm T. V. Khader N. . (2024). Lipid-associated macrophages' promotion of fibrosis resolution during MASH regression requires TREM2. Proc. Natl. Acad. Sci. U.S.A. 121, e2405746121. doi: 10.1073/pnas.2405746121 39172787 PMC11363294

[B19] Gao H. Jin Z. Bandyopadhyay G. Cunha E. R. K. Liu X. Zhao H. . (2022). MiR-690 treatment causes decreased fibrosis and steatosis and restores specific Kupffer cell functions in NASH. Cell Metab. 34, 978–990.e974. doi: 10.1016/j.cmet.2022.05.008 35700738 PMC9262870

[B20] Govaere O. Petersen S. K. Martinez-Lopez N. Wouters J. Van Haele M. Mancina R. M. . (2022). Macrophage scavenger receptor 1 mediates lipid-induced inflammation in non-alcoholic fatty liver disease. J. Hepatol. 76, 1001–1012. doi: 10.1016/j.jhep.2021.12.012 34942286 PMC7619241

[B21] Hasanoglu I. Rivero-Juárez A. Özkaya Şahin G. Escmid Study Group For Viral Hepatitis, E (2025). When metabolic dysfunction-associated steatotic liver disease meets viral hepatitis. J. Clin. Med. 14, 3422. doi: 10.3390/jcm14103422 40429417 PMC12112634

[B22] He C. Cheng D. Wang H. Wu K. Zhu Y. Lu N. (2018). Helicobacter pylori infection aggravates diet-induced nonalcoholic fatty liver in mice. Clin. Res. Hepatol. Gastroenterol. 42, 360–367. doi: 10.1016/j.clinre.2017.12.008 29657020

[B23] He S. Cui S. Song W. Jiang Y. Chen H. Liao D. . (2022). Interleukin-17 weakens the NAFLD/NASH process by facilitating intestinal barrier restoration depending on the gut microbiota. mBio 13, e0368821. doi: 10.1128/mbio.03688-21 35266816 PMC9040850

[B24] He J. Du C. Peng X. Hong W. Qiu D. Qiu X. . (2023). Hepatocyte nuclear factor 1A suppresses innate immune response by inducing degradation of TBK1 to inhibit steatohepatitis. Genes Dis. 10, 1596–1612. doi: 10.1016/j.gendis.2022.05.029 37397525 PMC10311033

[B25] Huang S. C. Kao J. H. (2022). Metabolic dysfunction-associated fatty liver disease and chronic hepatitis B. J. Formos Med. Assoc. 121, 2148–2151. doi: 10.1016/j.jfma.2022.07.013 35981929

[B26] Huang S. C. Liu C. J. (2023). Chronic hepatitis B with concurrent metabolic dysfunction-associated fatty liver disease: Challenges and perspectives. Clin. Mol. Hepatol. 29, 320–331. doi: 10.3350/cmh.2022.0422 36726053 PMC10121303

[B27] Hui R. W. Mak L. Y. (2025). MASLD after hepatitis C virus eradication: Do not overlook the cardiometabolic risk factors: Editorial on "Dynamic change of metabolic dysfunction-associated steatotic liver disease in chronic hepatitis C patients after viral eradication: A nationwide registry study in Taiwan. Clin. Mol. Hepatol. 31, 290–292. doi: 10.3350/cmh.2024.0630 39901340 PMC11791562

[B28] Iwata A. Maruyama J. Natsuki S. Nishiyama A. Tamura T. Tanaka M. . (2024). Egr2 drives the differentiation of Ly6C(hi) monocytes into fibrosis-promoting macrophages in metabolic dysfunction-associated steatohepatitis in mice. Commun. Biol. 7, 681. doi: 10.1038/s42003-024-06357-5 38831027 PMC11148031

[B29] Jung Y. Koo B. K. Jang S. Y. Kim D. Lee H. Lee D. H. . (2021). Association between circulating bile acid alterations and nonalcoholic steatohepatitis independent of obesity and diabetes mellitus. Liver Int. 41, 2892–2902. doi: 10.1111/liv.15030 34358397

[B30] Kamata Y. Kessoku T. Shimizu T. Sato S. Kobayashi T. Kurihashi T. . (2022). Periodontal treatment and usual care for nonalcoholic fatty liver disease: A multicenter, randomized controlled trial. Clin. Transl. Gastroenterol. 13, e00520. doi: 10.14309/ctg.0000000000000520 36000999 PMC10476832

[B31] Kan C. Zhang K. Wang Y. Zhang X. Liu C. Ma Y. . (2025). Global burden and future trends of metabolic dysfunction-associated steatotic liver disease: 1990-2021 to 2045. Ann. Hepatol. 30, 101898. doi: 10.1016/j.aohep.2025.101898 40057034

[B32] Kim H. Nelson P. Nzabarushimana E. Shen J. Jensen J. Bhosle A. . (2025). Multi-omic analysis reveals transkingdom gut dysbiosis in metabolic dysfunction-associated steatotic liver disease. Nat. Metab. 7, 1476–1492. doi: 10.1038/s42255-025-01318-6 40604156 PMC12320953

[B33] Kim T. J. Sinn D. H. Min Y. W. Son H. J. Kim J. J. Chang Y. . (2017). A cohort study on Helicobacter pylori infection associated with nonalcoholic fatty liver disease. J. Gastroenterol. 52, 1201–1210. doi: 10.1007/s00535-017-1337-y 28382402

[B34] Ko M. Y. Hsu Y. C. Yen H. H. Huang S. P. Su P. Y. (2025). The effects of pangenotypic direct-acting antiviral therapy on lipid profiles and insulin resistance in chronic hepatitis C patients. Viruses 17, 263. doi: 10.3390/v17020263 40007018 PMC11861053

[B35] Kralj D. Virović Jukić L. Stojsavljević S. Duvnjak M. Smolić M. Čurčić I. B. (2016). Hepatitis C virus, insulin resistance, and steatosis. J. Clin. Transl. Hepatol. 4, 66–75. doi: 10.14218/jcth.2015.00051 27047774 PMC4807145

[B36] Kuraji R. Ye C. Zhao C. Gao L. Martinez A. Miyashita Y. . (2024). Nisin lantibiotic prevents NAFLD liver steatosis and mitochondrial oxidative stress following periodontal disease by abrogating oral, gut and liver dysbiosis. NPJ Biofilms Microbiomes 10, 3. doi: 10.1038/s41522-024-00476-x 38233485 PMC10794237

[B37] Lang S. Demir M. Martin A. Jiang L. Zhang X. Duan Y. . (2020). Intestinal virome signature associated with severity of nonalcoholic fatty liver disease. Gastroenterology 159, 1839–1852. doi: 10.1053/j.gastro.2020.07.005 32652145 PMC8404510

[B38] Li Z. Gu R. Liu D. Zhang Y. Qin Y. Li X. . (2025). Lack of intestinal Mucin-1 impairs intestinal epithelial barrier and promotes metabolic dysfunction-associated steatotic liver disease in male mice. Nat. Commun. 17, 321. doi: 10.1038/s41467-025-67034-7 41350285 PMC12789075

[B39] Li J. Yang H. I. Yeh M. L. Le M. H. Le A. K. Yeo Y. H. . (2021). Association between fatty liver and cirrhosis, hepatocellular carcinoma, and hepatitis B surface antigen seroclearance in chronic hepatitis B. J. Infect. Dis. 224, 294–302. doi: 10.1093/infdis/jiaa739 33249474

[B40] Lin M. Gao B. Peng M. Chen X. Xiao H. Shi M. . (2024). Metabolic dysfunction-associated steatotic liver disease increases hepatocellular carcinoma risk in chronic hepatitis B patients: a retrospective cohort study. Front. Physiol. 15, 1347459. doi: 10.3389/fphys.2024.1347459 38405121 PMC10886697

[B41] Lin Z. Yang P. Hu Y. Xu H. Duan J. He F. . (2023). RING finger protein 13 protects against nonalcoholic steatohepatitis by targeting STING-relayed signaling pathways. Nat. Commun. 14, 6635. doi: 10.1038/s41467-023-42420-1 37857628 PMC10587083

[B42] Liu C. H. Chang Y. P. (2025). Metabolic dysfunction in patients following DAA-induced viral cure for HCV infection: A non-negligible risk to liver-related health: Editorial on "Adverse impact of metabolic dysfunction on fibrosis regression following direct-acting antiviral therapy: A multicenter study for chronic hepatitis C. Clin. Mol. Hepatol. 31, 658–661. doi: 10.3350/cmh.2025.0155 39957372 PMC12016587

[B43] Liu C. H. Cheng P. N. Fang Y. J. Chen C. Y. Kao W. Y. Lin C. L. . (2025). Risk of de novo HCC in patients with MASLD following direct-acting antiviral-induced cure of HCV infection. J. Hepatol. 82, 582–593. doi: 10.1016/j.jhep.2024.09.038 39368711

[B44] Liu J. Sun J. Yu J. Chen H. Zhang D. Zhang T. . (2023). Gut microbiome determines therapeutic effects of OCA on NAFLD by modulating bile acid metabolism. NPJ Biofilms Microbiomes 9, 29. doi: 10.1038/s41522-023-00399-z 37258543 PMC10232493

[B45] Liu Y. Xu H. Zhao Z. Dong Y. Wang X. Niu J. (2022). No evidence for a causal link between Helicobacter pylori infection and nonalcoholic fatty liver disease: A bidirectional Mendelian randomization study. Front. Microbiol. 13, 1018322. doi: 10.3389/fmicb.2022.1018322 36406444 PMC9669663

[B46] Loosen S. H. Kostev K. Luedde T. Roderburg C. (2023). Infectious mononucleosis is associated with an increased incidence of NAFLD. Eur. J. Clin. Invest. 53, e13911. doi: 10.1111/eci.13911 36409277

[B47] Luo Z. Ji Y. Zhang D. Gao H. Jin Z. Yang M. . (2022). Microbial DNA enrichment promotes liver steatosis and fibrosis in the course of non-alcoholic steatohepatitis. Acta Physiol. (Oxf) 235, e13827. doi: 10.1111/apha.13827 35500155 PMC9335517

[B48] Mantovani A. Morandin R. Fiorio V. Lando M. G. Gaviraghi A. Motta L. . (2025). Association between MASLD and increased risk of serious bacterial infections requiring hospital admission: A meta-analysis. Liver Int. 45, e16101. doi: 10.1111/liv.16101 39258758 PMC11892334

[B49] Mei T. Huang X. Tang S. Liu M. Zhang W. Yu H. (2024b). Effects of sustained viral response on lipid in hepatitis C: a systematic review and meta-analysis. Lipids Health Dis. 23, 74. doi: 10.1186/s12944-023-01957-2 38461262 PMC10924993

[B50] Mei E. H. Yao C. Chen Y. N. Nan S. X. Qi S. C. (2024a). Multifunctional role of oral bacteria in the progression of non-alcoholic fatty liver disease. World J. Hepatol. 16, 688–702. doi: 10.4254/wjh.v16.i5.688 38818294 PMC11135273

[B51] Moppert J. Domagalski K. Wrotek S. Pawłowska M. (2023). Are selected cytokines and Epstein-Barr virus DNA load predictors of hepatological complications of Epstein-Barr virus infection in children? J. Clin. Med. 12, 6158. doi: 10.3390/jcm12196158 37834802 PMC10573095

[B52] Papic N. Samadan L. Vrsaljko N. Radmanic L. Jelicic K. Simicic P. . (2022). Distinct cytokine profiles in severe COVID-19 and non-alcoholic fatty liver disease. Life. (Basel) 12, 795. doi: 10.3390/life12060795 35743825 PMC9225218

[B53] Sato S. Iino C. Furusawa K. Yoshida K. Chinda D. Sawada K. . (2025). Effect of oral microbiota composition on metabolic dysfunction-associated steatotic liver disease in the general population. J. Clin. Med. 14, 2013. doi: 10.3390/jcm14062013 40142822 PMC11943242

[B54] Sato S. Kamata Y. Kessoku T. Shimizu T. Kobayashi T. Kurihashi T. . (2022). A cross-sectional study assessing the relationship between non-alcoholic fatty liver disease and periodontal disease. Sci. Rep. 12, 13621. doi: 10.1038/s41598-022-17917-2 35948584 PMC9365789

[B55] Savage T. M. Fortson K. T. de Los Santos-Alexis K. Oliveras-Alsina A. Rouanne M. Rae S. S. . (2024). Amphiregulin from regulatory T cells promotes liver fibrosis and insulin resistance in non-alcoholic steatohepatitis. Immunity 57, 303–318.e306. doi: 10.1016/j.immuni.2024.01.009 38309273 PMC10922825

[B56] Shen R. Zhou Y. Zhang L. Yang S. (2023). The value of bile acid spectrum in the evaluation of hepatic injury in children with infectious mononucleosis caused by Epstein Barr virus infection. Front. Pediatr. 11, 1109762. doi: 10.3389/fped.2023.1109762 37025296 PMC10070945

[B57] Sohail A. Ali H. Patel P. Subramanium S. Dahiya D. S. Sohail A. H. . (2024). Impact of metabolic dysfunction-associated steatotic liver disease on COVID-19 hospitalizations: A propensity-matched analysis of the United States. World J. Virol. 13, 91149. doi: 10.5501/wjv.v13.i1.91149 38616849 PMC11008396

[B58] Targher G. Tilg H. Valenti L. (2025). Risk of serious bacterial and non-bacterial infections in people with MASLD. Liver Int. 45, e70059. doi: 10.1111/liv.70059 40072231 PMC11899495

[B59] Wang M. Li L. Qian J. Wang N. Bao J. Lu J. . (2023a). Periodontitis salivary microbiota exacerbates nonalcoholic fatty liver disease in high-fat diet-induced obese mice. iScience 26, 106346. doi: 10.1016/j.isci.2023.106346 36968080 PMC10031158

[B60] Wang Y. Wang Y. Duan G. Yang H. (2023b). NAFLD was independently associated with severe COVID-19 among younger patients rather than older patients: A meta-analysis. J. Hepatol. 78, e136–e139. doi: 10.1016/j.jhep.2022.10.009 36309129 PMC9597581

[B61] Wang L. Wang L. Liu M. Yuan Q. Cheng L. Chen H. . (2025). Characterization of the gut virome in patients with nonalcoholic fatty liver disease. J. Transl. Med. 24, 6. doi: 10.1186/s12967-025-07443-w 41316248 PMC12763994

[B62] Xu D. Qu X. Yang T. Sheng M. Bian X. Zhan Y. . (2024). The Foxo1-YAP-Notch1 axis reprograms STING-mediated innate immunity in NASH progression. Exp. Mol. Med. 56, 1843–1855. doi: 10.1038/s12276-024-01280-5 39122845 PMC11372114

[B63] Yang S. I. Geong J. H. Kim J. Y. (2014). Clinical characteristics of primary Epstein Barr virus hepatitis with elevation of alkaline phosphatase and γ-glutamyltransferase in children. Yonsei Med. J. 55, 107–112. doi: 10.3349/ymj.2014.55.1.107 24339294 PMC3874904

[B64] Yao C. Lan D. Li X. Wang Y. Qi S. Liu Y. (2023). Porphyromonas gingivalis is a risk factor for the development of nonalcoholic fatty liver disease via ferroptosis. Microbes Infect. 25, 105040. doi: 10.1016/j.micinf.2022.105040 35987459

[B65] Yu Y. Y. Tong Y. L. Wu L. Y. Yu X. Y. (2022). Helicobacter pylori infection eradication for nonalcoholic fatty liver disease: a randomized controlled trial. Sci. Rep. 12, 19530. doi: 10.1038/s41598-022-23746-0 36376474 PMC9663549

[B66] Zeng S. Schnabl B. (2024). Gut mycobiome alterations and implications for liver diseases. PloS Pathog. 20, e1012377. doi: 10.1371/journal.ppat.1012377 39116092 PMC11309506

[B67] Zhang C. Cui S. Mao G. Li G. (2021). Clinical characteristics and the risk factors of hepatic injury in 221 children with infectious mononucleosis. Front. Pediatr. 9, 809005. doi: 10.3389/fped.2021.809005 35096718 PMC8790314

[B68] Zhang X. Lau H. C. Ha S. Liu C. Liang C. Lee H. W. . (2025b). Intestinal TM6SF2 protects against metabolic dysfunction-associated steatohepatitis through the gut-liver axis. Nat. Metab. 7, 102–119. doi: 10.1038/s42255-024-01177-7 39779889 PMC11774752

[B69] Zhang P. Liu J. Lee A. Tsaur I. Ohira M. Duong V. . (2024). IL-22 resolves MASLD via enterocyte STAT3 restoration of diet-perturbed intestinal homeostasis. Cell Metab. 36, 2341–2354.e2346. doi: 10.1016/j.cmet.2024.08.012 39317186 PMC11631175

[B70] Zhang S. Mak L. Y. Yuen M. F. Seto W. K. (2025a). Mechanisms of hepatocellular carcinoma and cirrhosis development in concurrent steatotic liver disease and chronic hepatitis B. Clin. Mol. Hepatol. 31, S182–S195. doi: 10.3350/cmh.2024.0837 39568126 PMC11925439

[B71] Zhao M. Han X. Fan H. Liang C. Wang H. Zhang X. . (2025). Metabolic dysfunction-associated steatotic liver disease increases the risk of severe infection: A population-based cohort study. Liver Int. 45, e16136. doi: 10.1111/liv.16136 39422294

[B72] Zhao W. San J. Jiang F. Zhu Y. Wu G. Yang J. . (2026). Insulin-like growth factor 1 ameliorates intestinal barrier dysfunction in MASLD via IGF-1R/PI3K/AKT signaling. Nutrients 18, 1667. doi: 10.3390/nu18111667 42280311 PMC13257688

[B73] Zhao D. Yang F. Wang Y. Li S. Li Y. Hou F. . (2022). ALK1 signaling is required for the homeostasis of Kupffer cells and prevention of bacterial infection. J. Clin. Invest. 132, e150489. doi: 10.1172/jci150489 34874921 PMC8803331

[B74] Zheng N. Wang D. Xing G. Gao Y. Li S. Liu J. . (2025). Characterization of the gut mycobiome in patients with non-alcoholic fatty liver disease and correlations with serum metabolome. BMC Microbiol. 25, 660. doi: 10.1186/s12866-025-04348-y 41087898 PMC12522779

[B75] Zheng Y. Zhao L. Xiong Z. Huang C. Yong Q. Fang D. . (2024). Ursolic acid targets secreted phosphoprotein 1 to regulate Th17 cells against metabolic dysfunction-associated steatotic liver disease. Clin. Mol. Hepatol. 30, 449–467. doi: 10.3350/cmh.2024.0047 38623614 PMC11261229

[B76] Zhou X. Zhou D. Pu Y. Kim H. Sun Z. Qi W. . (2026). Multi-kingdom profiling reveals altered gut phage-bacteria-metabolite interactions in MASLD. Nat. Commun. 17, 5385. doi: 10.1038/s41467-026-71981-0 42000726 PMC13275893

